# Novel lipophosphonoxin-loaded polycaprolactone electrospun nanofiber dressing reduces *Staphylococcus aureus* induced wound infection in mice

**DOI:** 10.1038/s41598-021-96980-7

**Published:** 2021-09-03

**Authors:** Duy Dinh Do Pham, Věra Jenčová, Miriam Kaňuchová, Jan Bayram, Ivana Grossová, Hubert Šuca, Lukáš Urban, Kristýna Havlíčková, Vít Novotný, Petr Mikeš, Viktor Mojr, Nikifor Asatiani, Eva Kuželová Košťáková, Martina Maixnerová, Alena Vlková, Dragana Vítovská, Hana Šanderová, Alexandr Nemec, Libor Krásný, Robert Zajíček, David Lukáš, Dominik Rejman, Peter Gál

**Affiliations:** 1 Institute of Organic Chemistry and Biochemistry, Czech Academy of Sciences, Flemingovo nám. 2, 166 10 Prague 6, Czech Republic; 2 Faculty of Science, Humanities and Education, Technical University of Liberec, Studentska 1402/2, 461 17 Liberec 1, Czech Republic; 3 Laboratory of Cell Interactions, Center of Clinical and Preclinical Research MediPark, Pavol Jozef Šafárik University, Trieda SNP 1, 040 11 Košice, Slovak Republic; 4 Prague Burn Centre, Third Faculty of Medicine, University Hospital Královské Vinohrady, Šrobárova 50, 100 34 Prague 10, Czech Republic; 5 Department of Biomedical Research, East-Slovak Institute of Cardiovascular Diseases, Ondavská 8, 040 11 Košice, Slovak Republic; 6 The Institute for Nanomaterials, Advanced Technologies and Innovation, Bendlova 1409/7, 460 01 Liberec 1, Czech Republic; 7 Laboratory of Bacterial Genetics, Centre for Epidemiology and Microbiology, National Institute of Public Health, Šrobárova 49/48, 100 00 Prague 10, Czech Republic; 8 Unit for Biomedicine and Welfare of Laboratory Animals, National Institute of Public Health, Šrobárova 49/48, 100 00 Prague 10, Czech Republic; 9 Laboratory of Microbial Genetics and Gene Expression, Institute of Microbiology, Czech Academy of Sciences V.V.I., Vídeňská 1083, 142 20 Prague 4, Czech Republic; 10 Department of Medical Microbiology, Second Faculty of Medicine, Charles University, V Úvalu 84, 150 06 Prague 5, Czech Republic

**Keywords:** Biotechnology, Cell biology, Health care, Medical research

## Abstract

Active wound dressings are attracting extensive attention in soft tissue repair and regeneration, including bacteria-infected skin wound healing. As the wide use of antibiotics leads to drug resistance we present here a new concept of wound dressings based on the polycaprolactone nanofiber scaffold (NANO) releasing second generation lipophosphonoxin (LPPO) as antibacterial agent. Firstly, we demonstrated in vitro that LPPO released from NANO exerted antibacterial activity while not impairing proliferation/differentiation of fibroblasts and keratinocytes. Secondly, using a mouse model we showed that NANO loaded with LPPO significantly reduced the *Staphylococcus aureus* counts in infected wounds as evaluated 7 days post-surgery. Furthermore, the rate of degradation and subsequent LPPO release in infected wounds was also facilitated by lytic enzymes secreted by inoculated bacteria. Finally, LPPO displayed negligible to no systemic absorption. In conclusion, the composite antibacterial NANO-LPPO-based dressing reduces the bacterial load and promotes skin repair, with the potential to treat wounds in clinical settings.

## Introduction

Systemic use of antibiotics is currently the hallmark of treating (burn) wound infections, but it may result in harmful side-effects either through direct toxicity or by contributing to the emergence of resistant microorganisms^1,2^. Furthermore, many of the currently used topical antimicrobial agents exert cytotoxic effects on keratinocytes and fibroblasts, which may delay wound healing^3^. From this point of view, an efficient topical antimicrobial agent must act at a delicate balance between the need to control microbial growth in the wound bed and the potential risk that the agent impairs wound healing.

Previously, we reported the synthesis of novel compounds termed lipophosphonoxins (LPPO) exhibiting significant antibacterial activities against a wide range of bacteria, including multidrug-resistant strains, with no cytotoxicity on human cells at bactericidal concentrations^4–6^. LPPO act through permeabilization of the bacterial membrane leading to its disruption and cell death. So far, we have synthesized and tested two generations of LPPOs. The first LPPO generation^4^ displayed significant antibacterial activities against Gram-positive pathogens. Further modifications led to the second LPPO generation with improved antibacterial activities against both Gram-positive and Gram-negative bacteria^6^. Consequently, selected second generation LPPO have been successfully evaluated as antibacterial additives to bone cement^7^.

Conventional wound dressings such as gauze, polymer bandage or cotton wool, cannot prevent wound beds from drying and bacterial infection, and need to be replaced, which often results in damage of the healing tissue. Therefore, the development of an active antibacterial and repair/regeneration promoting (e.g. by stem/progenitor cells or growth factor), ideally biodegradable wound dressing is the key for further therapy towards normal healing in a fully controlled manner^8–11^. Faced with enormous quantities of clinically approved wound dressings it is tempting to find those with the most reliable clinically-relevant properties^12^. In this context, the polycaprolactone (PCL)-based nanofibrous scaffold wound dressing produced by electrospinning is one of the most promising materials as it mimics the original architecture of the native extracellular matrix (ECM) and is biodegradable, degraded into naturally occurring metabolite 6-hydroxyhexanoic acid. It also maintains appropriate moisture and supports gas exchange at the injury site^13^. Moreover, the PCL scaffold has been approved by the American Food and Drug Administration (FDA) for biomedical applications, such as a surgical suture and/or endodontic material^14,15^.

Microorganisms can develop resistance to conventional antimicrobial drugs. However, based on the structure and underlying mechanism of action, different from that of clinically used antibiotics, it is less likely that microbes will develop cross-resistance to LPPO^6^. Since electrospun nanofibrous scaffolds are potentially useful in medical applications such as wound healing and drug delivery^16^, we loaded the PCL-based wound dressing with LPPO to open new horizons towards novel approaches controlling wound infection. Our new concept was validated on a modified mice model of *Staphylococcus aureus* impaired wound healing and a series of in vitro experiments conducted on keratinocytes and fibroblasts.

## Results

### Wound dressing preparation and morphology

**Figure 1:**
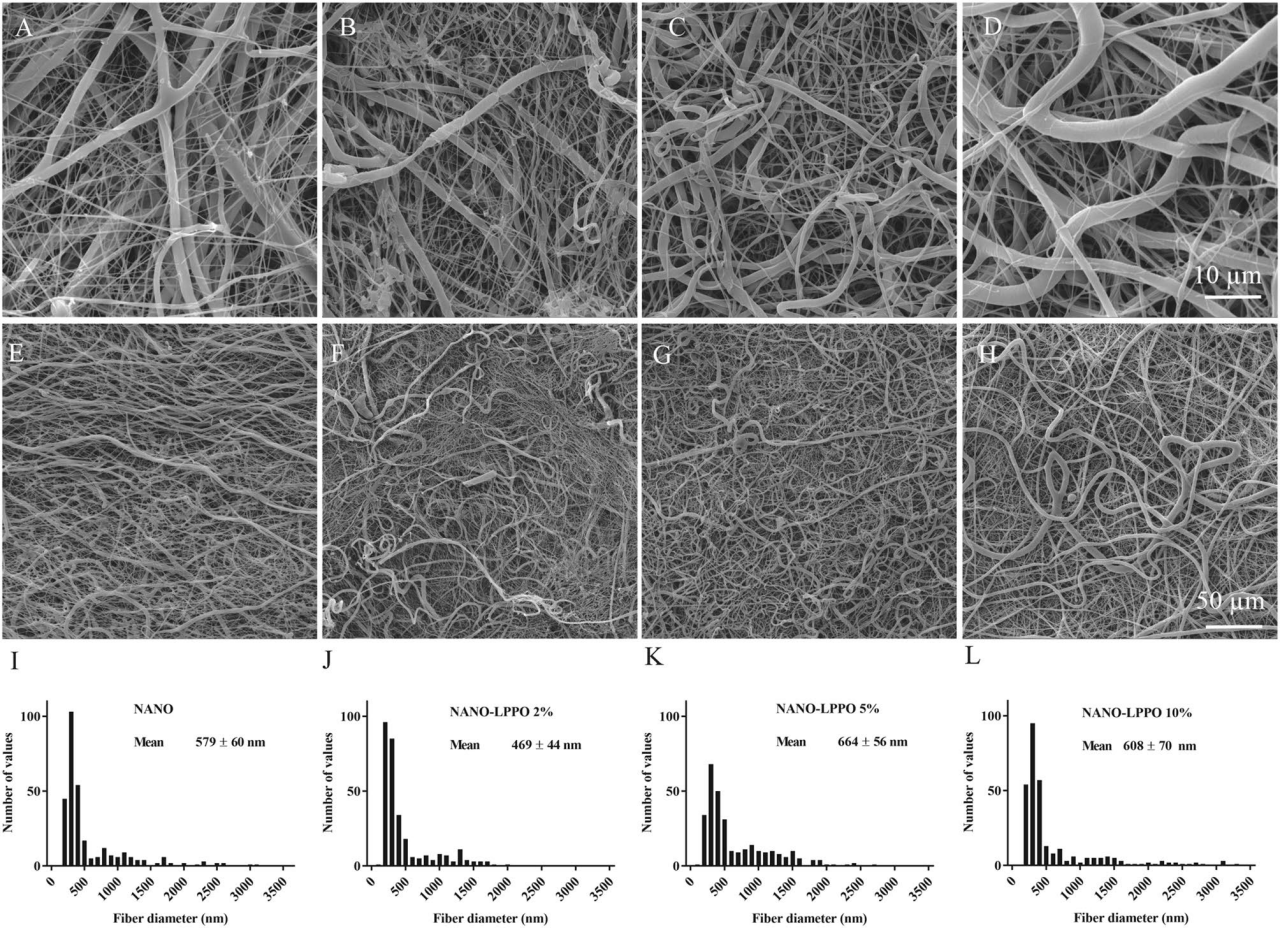
Representative photomicrographs from scanning electron microscope (**A**–**H**) and corresponding fiber diameter analysis (histograms, mean ± 95% confidence interval, n = 300) of electrospun PCL material (**I**–**L**) containing different amount of LPPO: 0% (**A**,**E**,**I**), 2% (**B**,**F**,**J**), 5% (**C**,**G**,**K**) and 10% (**D**,**H**,**L**). Scale bar top panel—10 µm (magnification 5000×), bottom panel—50 µm (magnification 1000×).

PCL-based nanofibrous wound dressings were prepared by blending electrospinning technology^17,18^, with the addition of increasing concentrations (0, 2, 5, and 10 wt%) of LPPO DR-6180 (hereafter LPPO). LPPO was selected for its strong antibacterial properties and low cytotoxicity and was synthesized according to the procedure published previously^6^. The quality of the prepared nanofibrous materials was assessed by scanning electron microscopy (SEM). SEM of scaffolds with subsequent basic image analysis of fiber diameters revealed comparable morphology of prepared materials (Fig. 1). Histograms show that the fiber diameters were 579 ± 60 nm for PCL, 469 ± 44 nm for NANO-LPPO2%, 664 ± 56 nm for NANO-LPPO5%, and 608 ± 70 nm for PCL-LPPO10%.

### LPPO release in PBS and LPPO release during enzymatically catalyzed degradation

**Figure 2:**
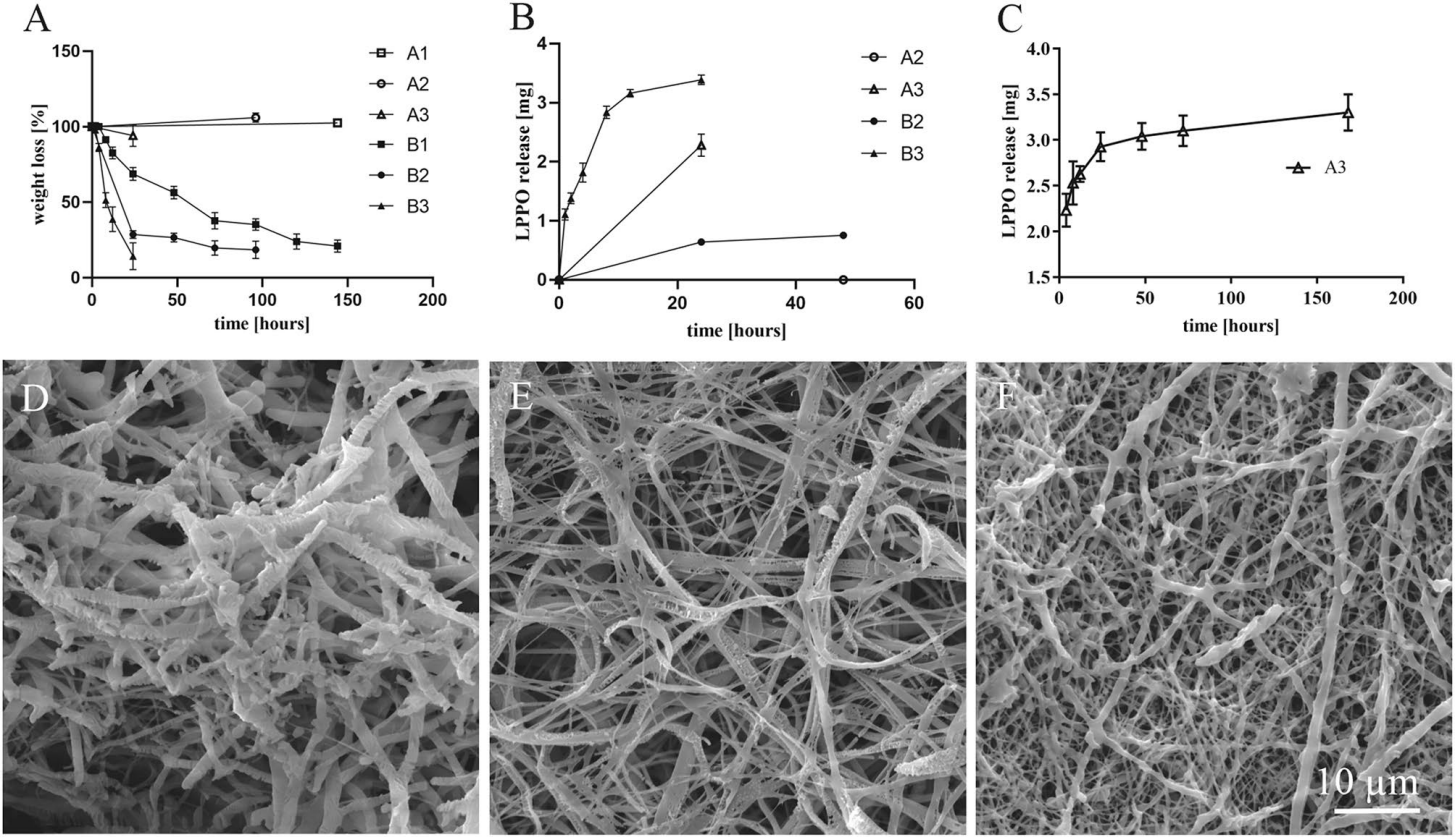
Graphs representing the degradation of NANO and cumulative release of LPPO (**A**–**C**) and SEM images of the materials (**D**–**F**). (**A**) weight loss of nanomaterials during incubation (A1–A3 without enzyme, B1–B3 in the presence of enzyme), (**B**) LPPO release in phosphate buffer saline within 1 week, (**C**) LPPO release during incubation (A2–A3 without enzyme, B2–B3 in the presence of enzyme), and. For (**B**) and (**C**): the total amount of LPPO released from 50 mg samples. A1/B1 NANO-LPPO0%, A2/B2 NANO-LPPO2%, A3/B3 NANO-LPPO10%. (**D**) SEM image of NANO at the 4th day of degradation, (**E**) SEM image of NANO-LPPO2% at the 3rd day of degradation, (**F**) SEM image of NANO-LPPO10% at the 1st day of degradation, Samples are labeled according to Table 1).

For the antibiotic function of the NANO-LPPO wound covers to be effective, controlled release of the active compound, LPPO, is necessary. Figure 2A shows a weight loss of NANO-LPPO samples incubated in PBS in the presence or absence of lipase. In a phosphate-buffered saline environment, only the material with the highest concentration of LPPO (NANO-LPPO10%) showed a strong release of LPPO (Fig. 2B). The LPPO release from NANO-LPPO10% occurred gradually, in the first 24 h 2.9 mg was released rapidly. This is the so-called burst release, which is typical for drugs incorporated using blending electrospinning technology^17,18^. Then the release continued more slowly and after 7 days the total amount of LPPO released was 3.3 mg. For NANO-LPPO2%, no detectable drug release was observed during the 7-day incubation in PBS.

We next monitored the LPPO release in the presence of a lipase. Lipases are ubiquitous enzymes capable of degradation of PCL-based nanomaterials present in humans and/or produced by bacteria. The lipase-catalyzed degradation assay (experiments B1–B3, Table 1) indicated that LPPO was released during NANO degradation in a concentration-dependent manner (Fig. 2C). Importantly, the release occurred even from NANO-LPPO2%, which was resistant to release in PBS. The release followed a two-step kinetic, with the most pronounced release during the first day (75%), followed by a reduced degradation rate during the remaining observation period. The total amount of LPPO released from 50 mg of the original sample (NANO-LPPO2%) was 0.75 mg. Interestingly, the NANO-LPPO10% was completely degraded within 24 h (50% was lost within the first 8 h). The total amount of LPPO released during the experiment from 50 mg of the original total sample weight of NANO-LPPO10% was 3.39 mg.

**Table 1:** Release experiments performed with NANO-LPPO in PBS with/o lipase.

Sample	Material	Lipase	Analysis
A1	NANO	−	Solution: LPPO release (HPLC)
A2	NANO-LPPO2%	−	Solution: LPPO release (HPLC)
A3	NANO-LPPO10%	−	Solution: LPPO release (HPLC)
B1	NANO	+	Solution: LPPO release (HPLC) remaining material: weight loss, SEM
B2	NANO-LPPO2%	+	Solution: LPPO release (HPLC) remaining material: weight loss, SEM
B3	NANO-LPPO10%	+	Solution: LPPO release (HPLC) remaining material: weight loss, SEM

*LPPO* lippophosphonoxin DR-6180, *NANO* polycaprolactone based nanofiber/wound dressing, *PBS* phosphate-buffered saline, *SEM* scanning electron microscopy.

The level of degradation was also reflected by distinct changes in the fiber morphology (Fig. 2D–F). When compared to the control sample (NANO without LPPO) (Fig. 2D) where structural changes occurred only on the surface of fibers, NANO-LPPO2% and NANO-LPPO10% (Fig. 2E,F) displayed more advanced stages of material degradation with broken fibers probably due to complete splitting of the polymer in its amorphous parts. The NANO-LPPO2% scaffolds were also characterized by the formation of web-like fiber structures (Fig. 2E), not detected in the NANO-LPPO10% material.

### PCL-based wound dressing wettability

We hypothesized that the rate of NANO-LPPO decomposition depends on the ability of LPPO to alter nanofiber surface wettability. Therefore, the wound dressing wettability, an important parameter affecting the rate of fluid absorption and moisture control was assessed using the Washburn adsorption test. The experiments revealed that the presence of LPPO in PCL nanomaterial dramatically increased the wettability of the nanofiber (Fig. S1). The Washburn method^19,20^ revealed faster initial weight gain (higher wicking rate) for PCL loaded with LPPO (NANO-LPPO10%). The final slope of line at *y* = 0.215 ± 0.003 g^2^ s^−1^ and regression coefficient at *R* = 0.83 ± 0.07 were measured for NANO-LPPO10% samples. On the other hand, the slope of the line for the negative control was significantly smaller at *A* = 0.0003 ± 0.00015 g^2^ s^−1^ with the regression coefficients at *R* = 0.96 ± 0.03.

### Effect of LPPO on HDFs and HaCaTs

Since the antibacterial activity of the NANO-LPPO wound dressing is based on the controlled release of LPPO from the nanofibrous scaffold, we were interested in whether LPPO at bactericidal concentrations modulates proliferation/differentiation of fibroblasts and keratinocytes. Furthermore, we also investigated the ability of LPPO to interfere with TGF-1 signaling that is important for proper wound closure^21^.

### Effect of LPPO on cell proliferation

**Figure 3:**
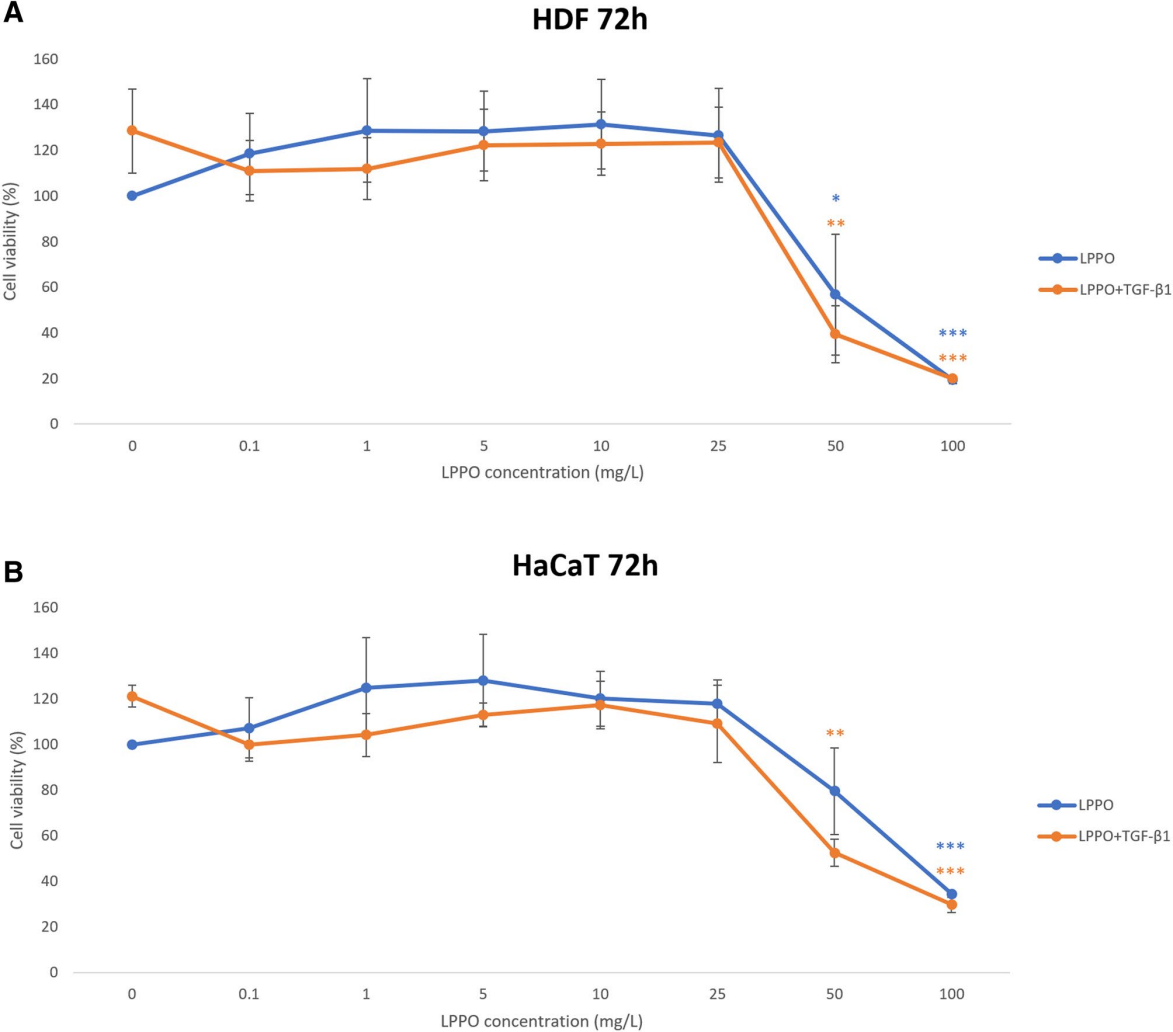
MTS-assay of human dermal fibroblasts (HDFs) (**A**) and HaCaT keratinocytes (**B**) cultured in the presence of LPPO and/or TGF-β1 (30 ng/mL) for 72 h (*p < 0.05; **p < 0.01; ***p < 0.001).

The in vitro investigation conducted on HDFs (Fig. 3A) and HaCaT (Fig. 3B) cells revealed that LPPO in the concentration range from 0.1 to 25 mg/L (bactericidal concentrations) does not impair the metabolic activity of studied cells. Of note, higher tested concentrations of LPPO, 50 and 100 mg/L, were found toxic to studied cells.

### Effect of LPPO on cell migration

**Figure 4:**
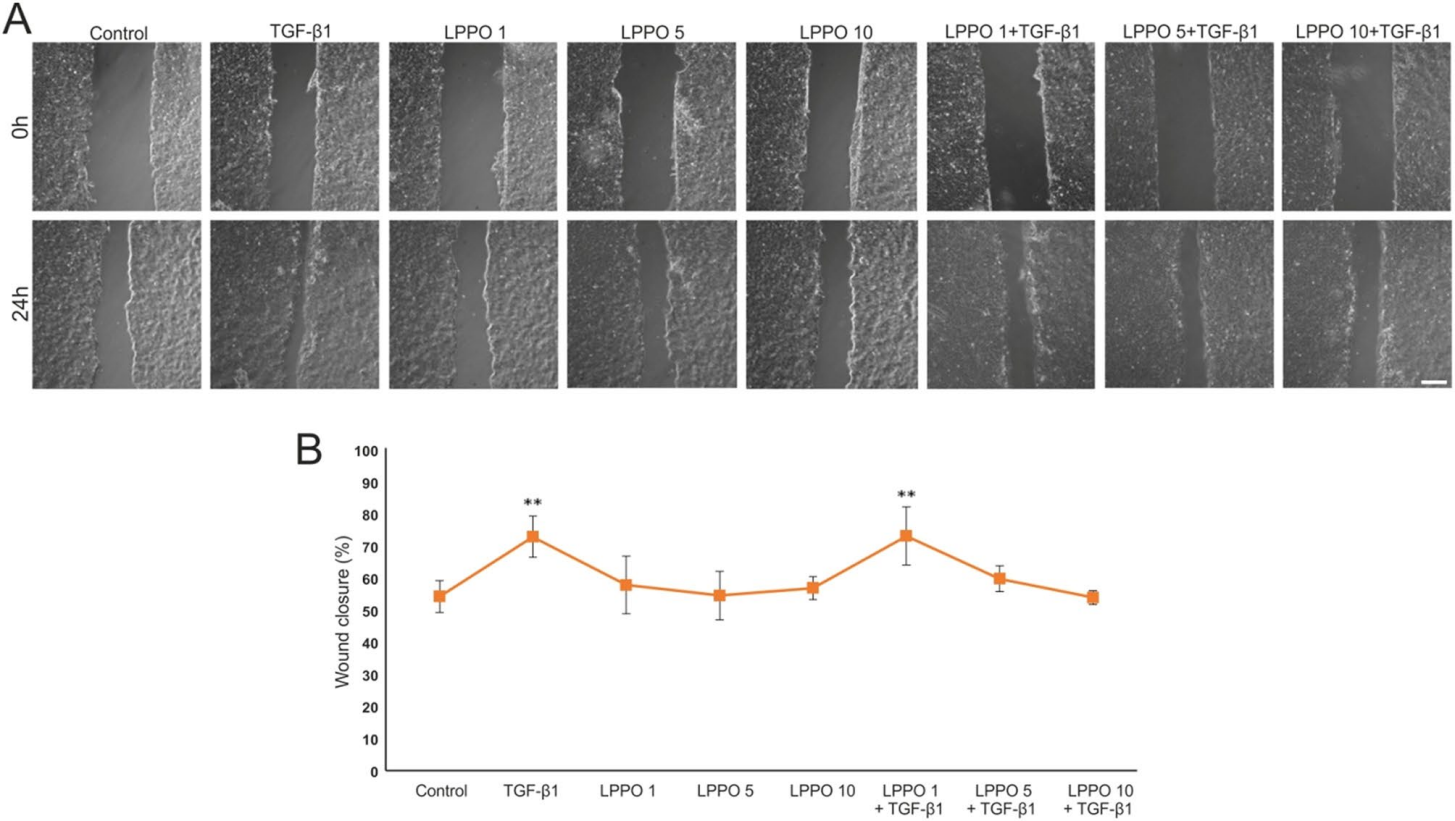
Migration (2D wound healing) assay of HaCaT keratinocytes with LPPO (1, 5 and 10 mg/mL) and/or TGF-β1 (30 ng/mL); (**A**) representative figures of scratched confluent layer of HaCaT cells at time 0 and following 24 h; (**B**) Mean ± SD values from three independent experiments (**p < 0.01) (scale bar = 200 µm).

It is well known that keratinocyte migration is essential for proper wound reepithelization, thus we examined how LPPO affects 2D wound healing in HaCaT cells. As illustrated in Fig. 4 keratinocytes migration was accelerated in the presence of TGF-1β (positive control). When compared to the control, LPPO (at any of the tested concentrations) did not significantly change the migratory ability of cells. However, LPPO attenuated TGF-1β-induced migration at higher tested concentrations of 5 and 10 mg/L, but not when added at 1 mg/L.

### Western blot of HDFs and HaCaTs following treatment with LPPO

**Figure 5:**
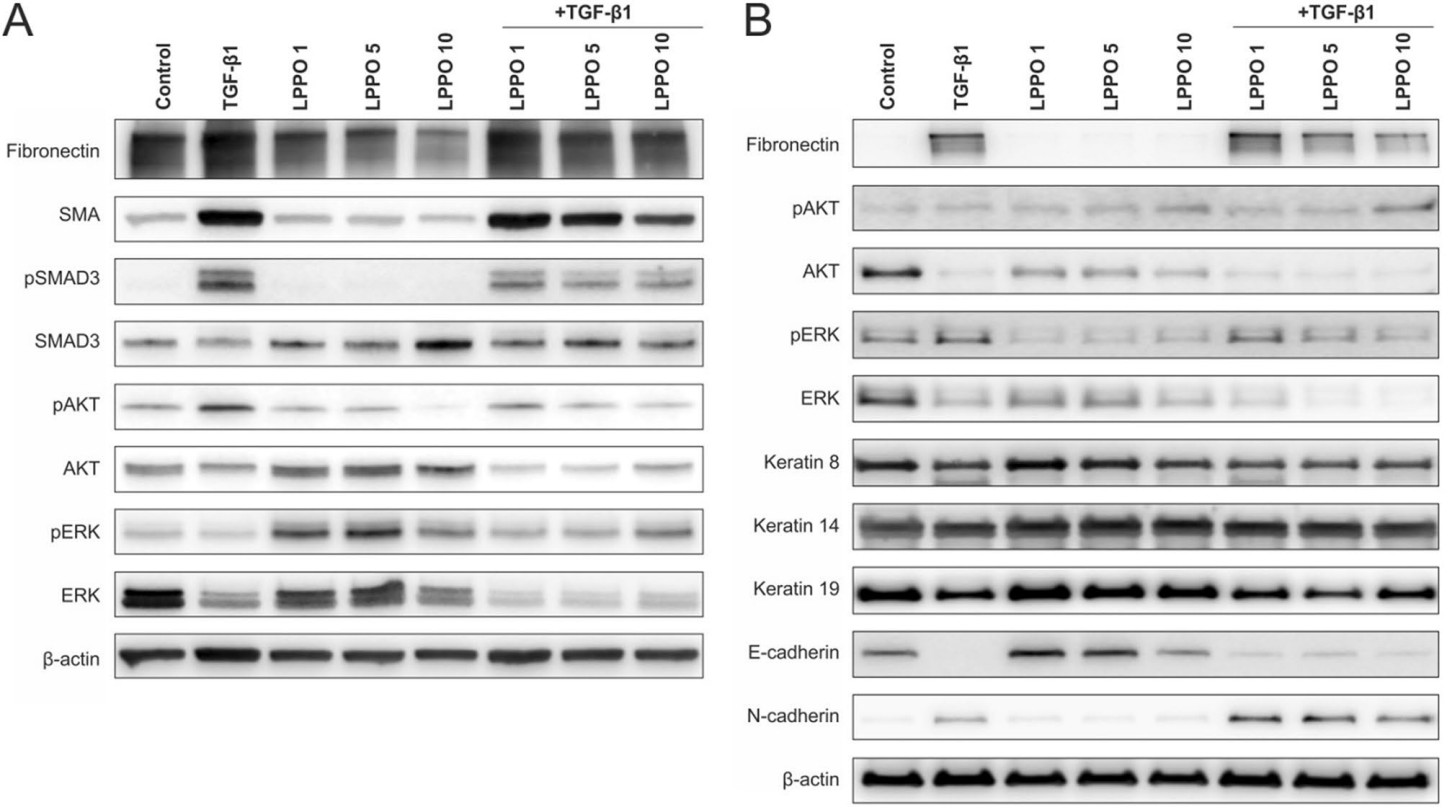
(**A**) Western blot analysis of LPPO and TGF-β1 30 ng/mL modulation of primary culture of human dermal fibroblasts (full-length gel is shown in Fig. S2). (**B**) Western blot analysis of LPPO and TGF-β1 (30 ng/mL) modulation of human keratinocyte cell line HaCaT (full-length gel is shown in Fig. S3).

Results from the Western blot (WB) analysis are shown in Fig. 5. As observed in HDFs (Fig. 5A), TGF-β1 activated the canonical (SMAD) signaling resulting in stimulated production of SMA and fibronectin. Similarly, TGF-β1 increased pAKT expression whereas the expressions of pERK/ERK were rather slightly decreased. HDFs treatment with LPPO, in the presence or absence of TGF-β1, slightly decreased fibronectin and SMA expressions in a concentration-dependent manner. In this context, the TGF-β1-induced canonical signaling was also slightly attenuated with increasing concentration of LPPO. A similar picture was seen in the trend of pAKT expression while total AKT was rather stimulated with LPPO. On the other hand, LPPO activated the non-canonical (pERK) signaling, but this effect was abolished in the presence of TGF-β1.

In parallel, HaCaT phenotype (Fig. 5B) was characterized by no production of fibronectin, expression of AKT/ERK total forms (absence of pAKT, poor expression of pERK), marked expression of keratins-8, -14, and -19 as well as the presence of E-cadherin and absence of N-cadherin. TGF-β1 treatment of keratinocytes resulted in fibronectin expression unchanged pAKT and decreased total AKT production as well as increased pERK levels and to E- to N-cadherin switch. The keratin profile remained rather stable. LPPO slightly stimulated pAKT, but reduced total AKT and pERK/ERK levels. Moreover, E-cadherin expression was stimulated at lower tested concentrations of LPPO whereas the highest tested concentration decreased E-cadherin expression. Importantly, LPPO did not remarkably interfere with phenotypic changes induced by TGF-β1.

### Immunofluorescence of HDFs and HaCaTs following treatment with LPPO

**Figure 6:**
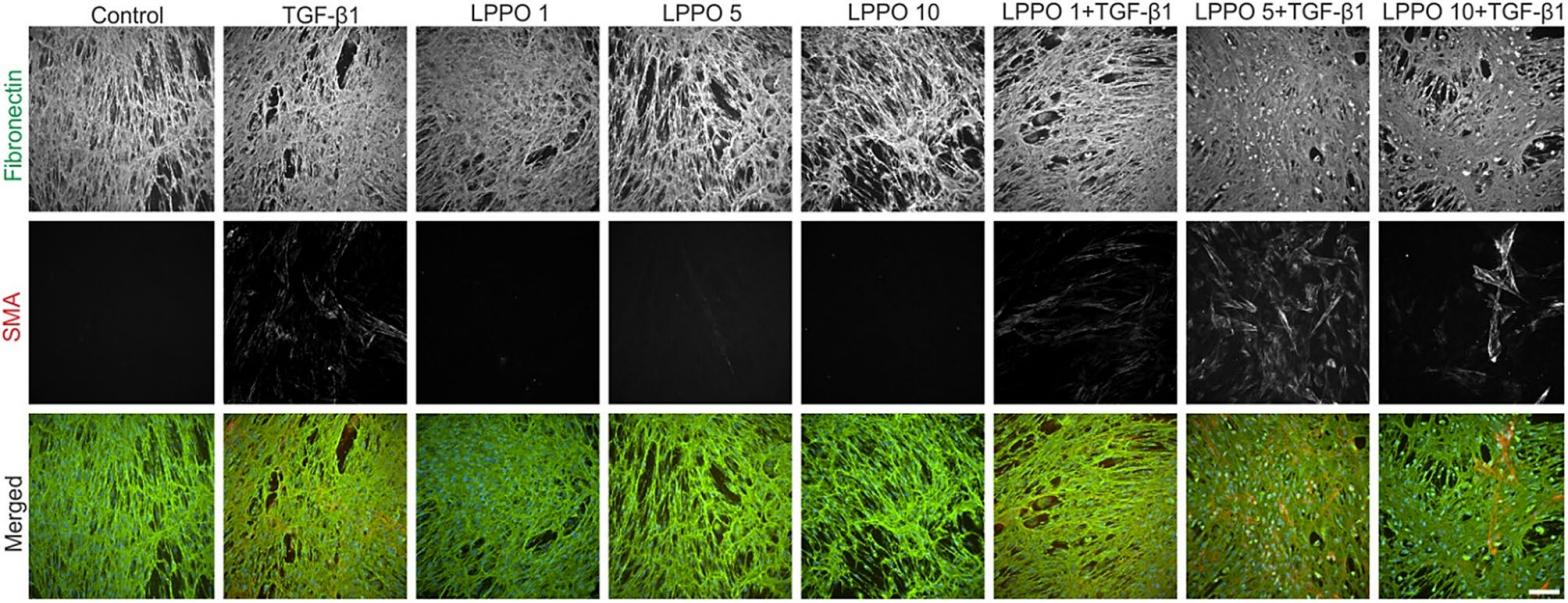
Immunofluorescent analysis of cytokeratins-8, 14 and 19 expression in human dermal fibroblasts cultivated with LPPO (1, 5, and 10 mg/L) in presence or absence of TGF-β1 (30 ng/mL) (scale bar = 100 µm).

Immunofluorescence staining of fibronectin showed that HDFs (Fig. 6) form ECM scaffold, which is more fibronectin-dense following TGF-β1 treatment. As expected, TGF-β1 also stimulated differentiation of HDFs into myofibroblasts (with well-formed SMA stress fibers). LPPO did not remarkably reduce fibronectin deposition nor induced SMA expression. Importantly, the TGF-β1-induced ECM and SMA production were not affected by LPPO at 1 and 5 mg/L. Of note, LPPO at 10 mg/L slightly decreased ECM synthesis and TGF-β1-induced ECM and SMA depositions.

**Figure 7:**
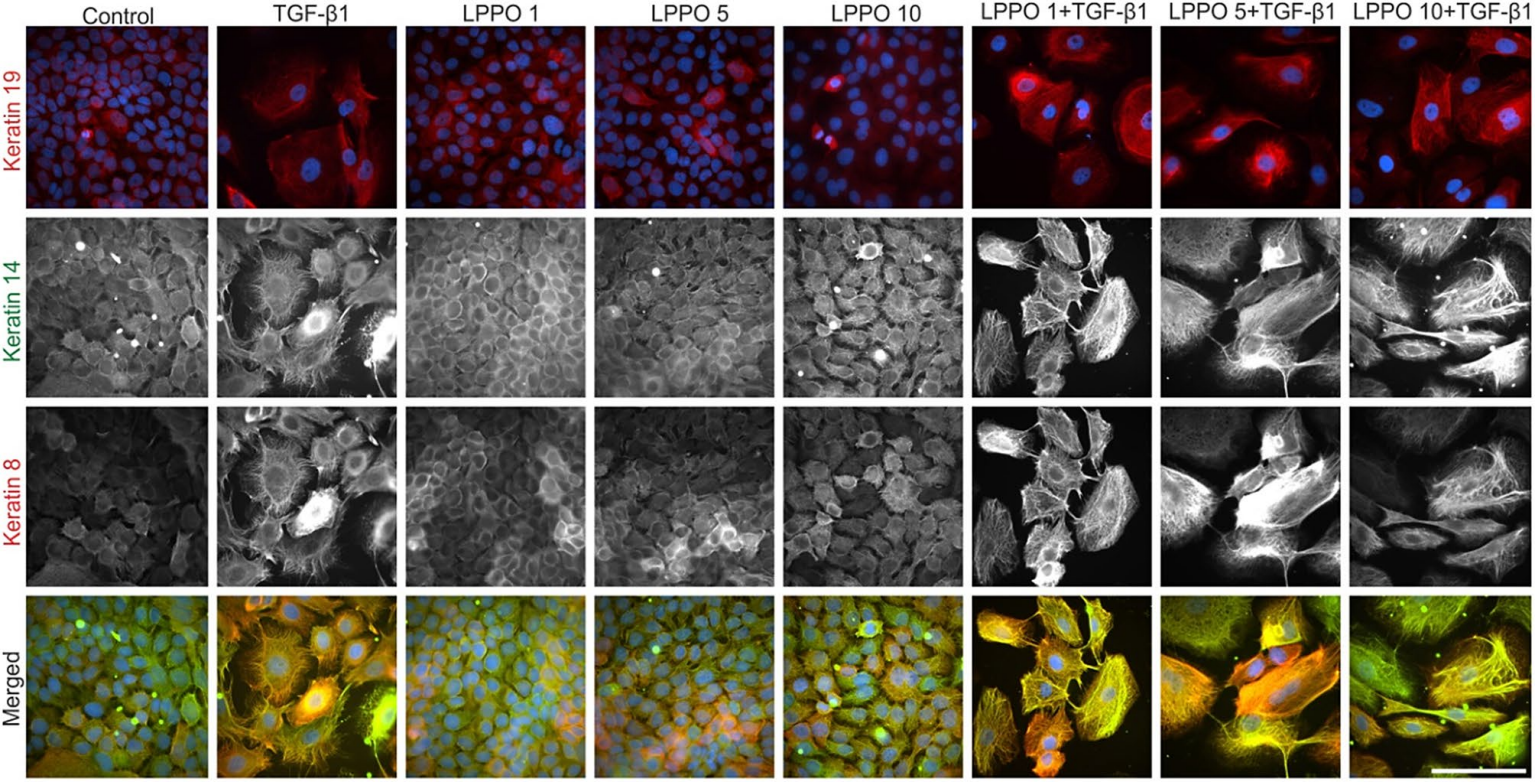
Immunofluorescent analysis of cytokeratins-8, 14 and 19 expression in HaCaT cells cultivated with LPPO (1, 5, and 10 mg/L) in presence or absence of TGF-β1 (30 ng/mL) (scale bar = 100 µm).

The morphology of HaCaT cells (Fig. 7) was clearly different in cells treated with TGF-β1 (large cells) when compared to the control (small cells). Untreated cells expressed moderate levels of keratins 14 and 19 while the expression of keratin 8 was minimal. Both, TGF-β1 and LPPO slightly increased keratin 8 levels.

### Mice model

Next, we demonstrated that used animal model is suitable to study the clinically relevant *S. aureus* as wound healing impairing pathogen and NANO-LPPO as an efficient antibacterial wound dressing. The wound experiment was evaluated on post-operative day 7. In detail, wound infection was clearly visible by the presence of purulent exudate (not shown). NANO-LPPO5%- and NANO-LPPO10%-based wound dressings displayed strong infection-impairing properties whereas NANO-LPPO2% exhibited only limited activity. Subsequently, the animal experiment was evaluated microscopically (see “Histology of open wounds” section) as well as using agar-based culture (see “Cultivation and characterization of bacteria” section) and PCR-based quantification of *S. aureus* presence in wounds and surrounding skin (see “Bacteria qPCR-based quantification in wounds” section).

### Histology of open wounds

**Figure 8:**
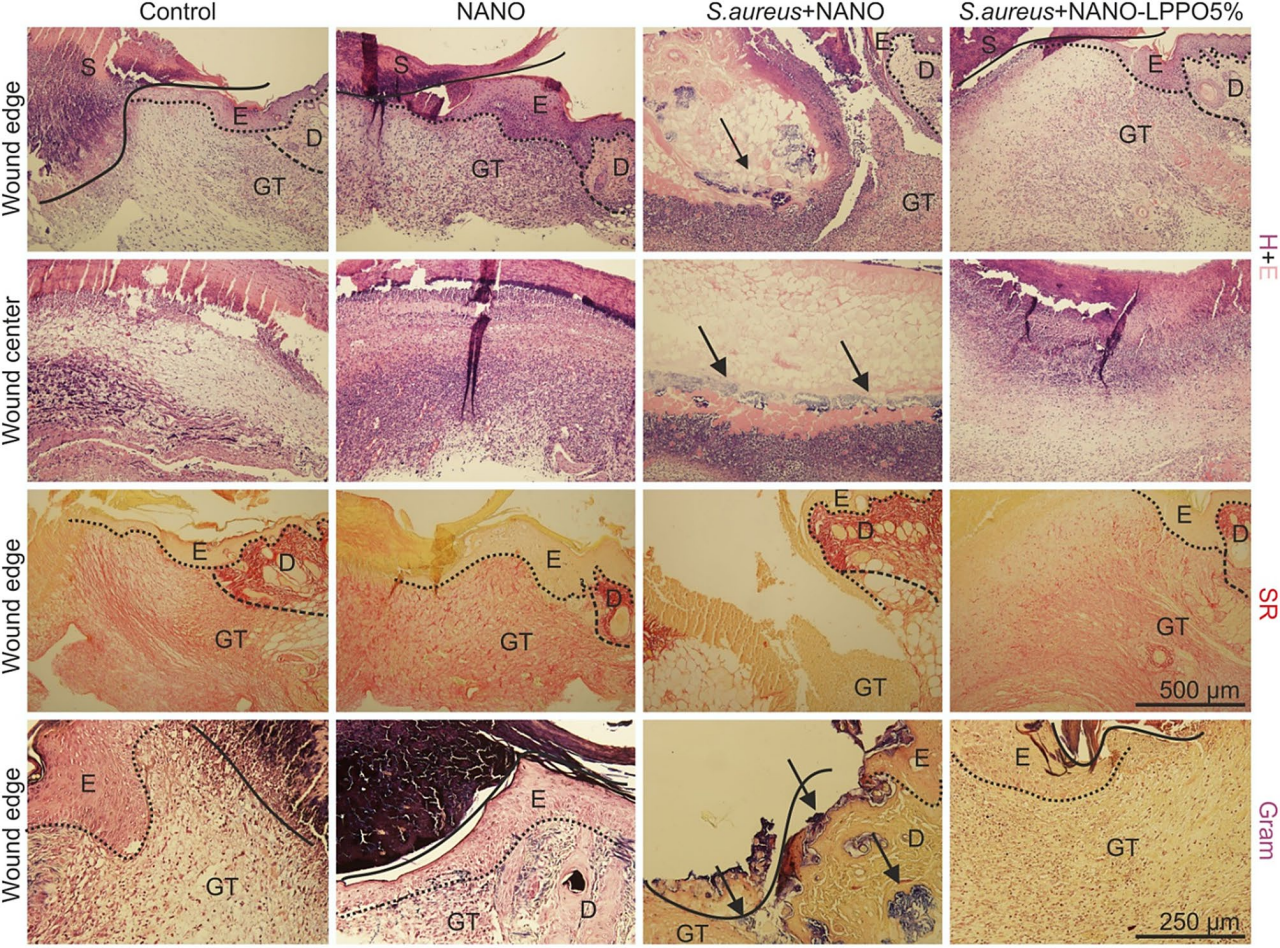
Histological photographs of skin wounds at day 7 post-surgery. Sections were stained with hematoxylin–eosin (H + E; arrows show the presence of bacterial infection), Sirius red (SR) and Gram (arrows show the presence of gram-positive bacterial infection) staining (*D* dermis, *E* epidermis, *GT* granulation tissue, *S* scab; scale 250 µm).

For the histological analysis we used hematoxylin and eosin, Sirius red (collagen) and Gram (gram-positive bacteria) staining; the results are shown in Fig. 8.

First, the effect of NANO-LPPO on the healing of non-infected mice was examined. On day seven, the demarcation line (dominantly formed by neutrophils) separated vital tissue from necrosis, thus the acute inflammatory phase was almost finished. The epithelial sheet migrated beneath the scab, but did not reach the central portion of the wounds. The newly formed granulation tissue (GT) was populated by fibroblasts and luminized vessels. However, GT was well-formed only at wound edges whereas the central parts of wounds were filled only with provisional wound matrix. The control NANO dressing slightly improved granulation tissue formation that was seen also in the central parts of wounds while other parameters remained rather stable. The use of NANO-LPPO-based (at any of the tested LPPO concentration) wound dressing did not alter the morphological dynamics of examined parameters.

Next, histological analysis of *S. aureus* infected wounds revealed the known capacity of this bacterium to impair the healing process. In a qualitative comparison to non-infected control, infection led to persisting infiltration of injury site with inflammatory cells, re-epithelization restricted to a thickening of cut edges of the epidermis, remarkably decreased numbers of fibroblasts and luminized vessels with an absence of newly formed collagen in the granulation tissue. A similar picture was detected in wounds treated with LPPO at 2%. Importantly, an increase in re-epithelization, granulation tissue formation, collagen score was observed at this time point for LPPO 5% and 10% with no remarkable differences from the uninfected control.

### Cultivation and characterization of bacteria

The next approach was the inspection of bacteria from wound swabs grown on agar media. Agar cultures were first inspected visually, then streaked on agar plates, and the resulting colonies, representing different morphotypes, were identified by MALDI-TOF MS (Table S1). *Staphylococcus aureus* CNCTC 5480 was detected in all samples without LPPO (S31–S36) or with 2% LPPO (S31–S36). In contrast, samples containing 5% (S43–S48) and 10% (S49–S54) LPPO yielded no visible growth of *S. aureus* CNCTC 5480, with the only exception of S48. Moreover, visual inspection revealed that samples with 2% LPPO produced reduced growth of *S. aureus* as compared with the samples without LPPO. Some of the BA plates with either control samples (S1–S30) or inoculated samples containing 5–10% LPPO (S43–S54) yielded sporadic colonies of non-*S. aureus* bacteria such as *Staphylococcus sciuri*, *Enterococcus gallinarum* or unspecified alfa-hemolytic streptococci. All these bacteria are known as potential colonizers of mammalian skin, with *S. sciuri* being commonly isolated from laboratory mice. In addition, control samples S7–S10 and inoculated samples S37 and S48 yielded colonies of a strain of *S. aureus*, which was both phenotypically and genotypically distinct from *S. aureus* CNCTC 5480. The most likely source of the bacteria other than *S. aureus* CNCTC 5480 was prior skin colonization of experimental mice and/or cross-contamination occurring during the experiment. In any case, the presence of the non-*S. aureus* CNCTC 5480 bacteria did not interfere with the interpretation of the results.

### Bacteria qPCR-based quantification in wounds

**Figure 9:**
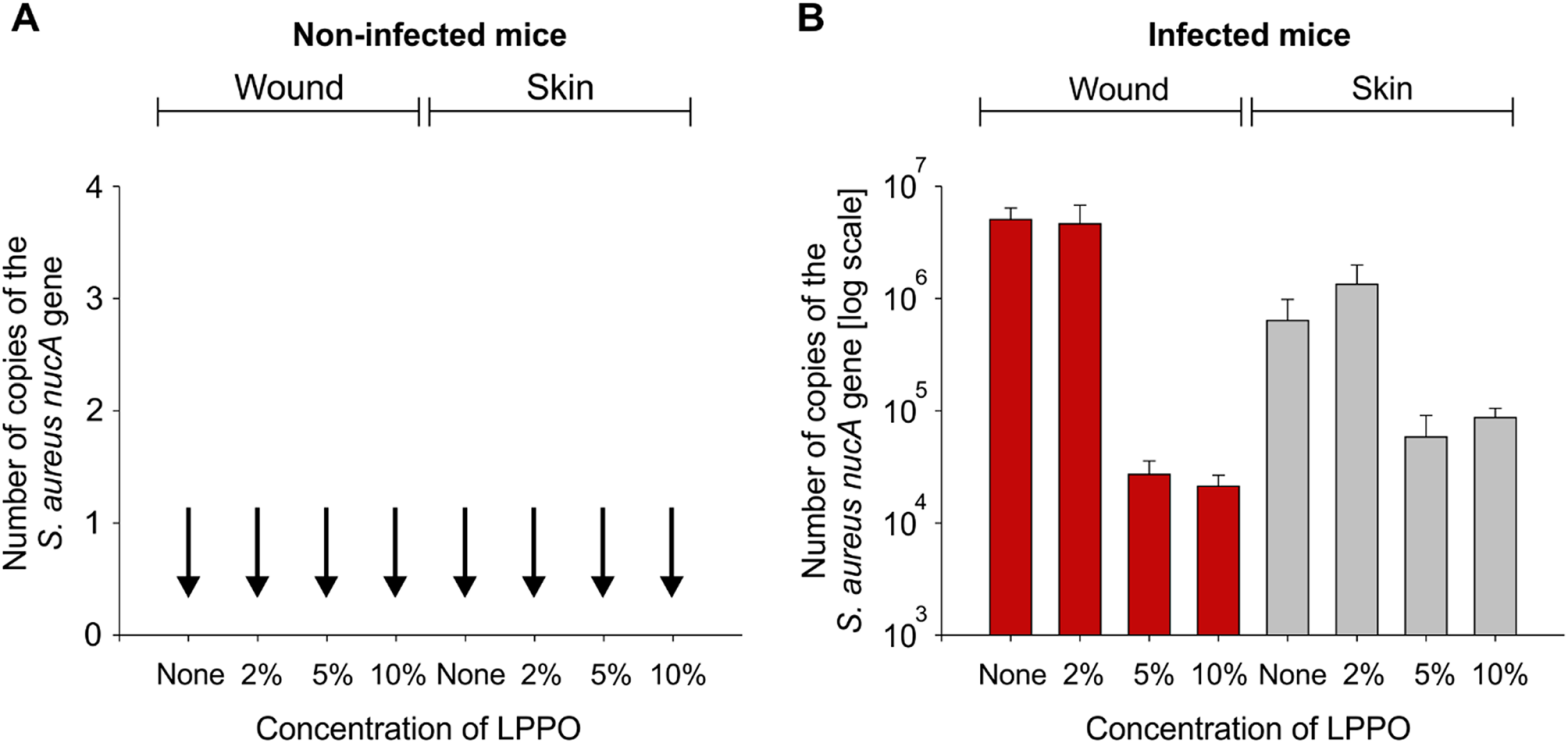
LPPOs prevent the growth of *S. aureus* in mice skin tissue wounds. (**A**) qPCR results from non-infected mice. The number of copies of the *nucA* gene was used as a proxy for the number of bacteria. The vertical arrows indicate zero values. (**B**) qPCR results from infected mice (wounds and surrounding skin tissue). Red bars, numbers of bacteria from wounds; grey bars, numbers of bacteria from the skin surrounding the wound. The presence/absence of LPPOs is indicated below the graph. The bars show averages from three to four animals, the error bars show ± SD.

The third approach was qPCR-based quantitation of *S. aureus* in the wound and surrounding skin. The results are shown in Fig. 9. From the qPCR data, it was apparent that the bacterial load in the wounds covered with NANO-LPPO5% and 10% compared to NANO and NANO-LPPO2% was reduced by several orders of magnitude, correlating with the findings from the histological and bacteriological analyses. We note that qPCR can detect also DNA releases from dead bacteria. Hence, the actual load of viable bacterial cells was likely lower than the apparent values obtained by qPCR.

### Determination of LPPO in mouse plasma and liver by LC–MS/MS

Finally, to evaluate whether LPPO can be after its topical application absorbed and systemically distributed, we analyzed samples of blood and liver homogenates for its presence. As shown in Tables S2 and S3, the amounts of LPPO detected in blood and liver correlated with its amounts in the NANO-LPPO composite. However, the detected systemic LPPO levels were negligible from the cytotoxicity point of view^6^.

## Discussion

In the present study, we developed and successfully tested a new electrospun PCL-based scaffold loaded with antibacterial LPPO molecule (NANO-LPPO) promoting healing in a standardized murine model of *S. aureus* induced wound infection. The biocompatible and biodegradable scaffold consists of a skin ECM-like^22^ macroporous network of cross-linked smooth fibers (diameter around 1 µm, pores of several micrometers), with the ability to extract wound exudate and deliver oxygen^23^. This biodegradable material provides inherent adhesion and occlusion, integrates into the wound and reduces the rate of dressing replacement^24^. Scaffolds loading with LPPO did not affect fiber size but enhanced wettability and biodegradation and provided significant antibacterial properties.

The degradation of PCL started as light surface modifications and continued from day 2 in significant restructuring of the entire fiber. In detail, fiber morphology changed due to the rearrangement of macromolecules as a result of the formation of regions with crystalline and amorphous structure^25,26^. Interestingly, restructuring was more pronounced when PCL was loaded with LPPO^27^ as demonstrated by crushed/broken fibers due to the complete splitting of the polymer in the amorphous regions. In this context, we may hypothesize at least two basic mechanisms by which LPPO enhances NANO degradation. Firstly, the mechanical properties of NANO could be directly altered by the presence of LPPO molecule. Secondly, LPPO, as an amphiphilic molecule, acts as a surfactant and thus decreases the hydrophobicity of NANO by facilitating the access of the hydrolytic enzymes to a larger surface of the NANO-fiber. Molecular interactions between the hydrophobic parts of NANO and LPPO may lead to changes in the fiber structure enhancing the diffusion of enzymes into the material. Therefore, decreasing hydrophobicity of NANO in the presence of LPPO has been verified via measurement of the wettability. As expected NANO-LPPO10% exhibited significantly better wettability when compared to the control (hydrophobic pure PCL material).

LPPO release from NANO demonstrated a dose-dependent pattern in PBS. Whereas the release of LPPO10% occurred gradually by the so-called burst release (typical for drugs incorporated using blending electrospinning technology^17,18^) followed by a decrease of the release rate, LPPO2% was not released during the 7-day incubation period. It appears that the release of LPPO by simple diffusion occurs only at higher concentrations of the incorporated LPPO. This correlates with our previous findings where the elution kinetics of LPPO from polymethyl methacrylate cement (used in orthopedic surgery) resembled the elution of well-known antibiotics^7,28^, supporting the clinical potential of the proposed dressing.

Interestingly, enzymatically catalyzed degradation of NANO revealed a positive correlation of the degradation rate with the amount of loaded LPPO molecule. An initial spike was observed within a few hours after the application, followed by a more gradual prolonged elution in the order of days. This aligns well with the potential therapeutic use, where the elevated initial concentration kills existing bacteria, and this is followed by the elution of decreased, but still sufficient, amounts of the compounds providing the antibacterial milieu for an extended time^29,30^. Moreover, our experiment also indicates that LPPO release is also stimulated by the presence of bacteria in the wound by increasing the lipase activity in the presence of *S. aureus*^31^.

Current in vitro experiment was conducted to reveal whether LPPO that is released from NANO during the degradation of PCL impairs selected biological processes associated with wound repair. LPPO impaired neither the proliferation of human dermal fibroblasts nor TGF-β1-induced fibroblast-to-myofibroblast transition or fibronectin-rich ECM formation. In particular, the pro-fibrotic cytokine (TGF-β1) is transiently up-regulated in normal skin wounds where its ability to induce myofibroblasts is relevant for wound closure^32^. The LPPO ability to interfere with TGF-β1 signaling, both canonical TGF-β1/SMAD and non-canonical/non-SMAD (MAPK, PI3K)^33^, was not observed in our experiment. We further tested whether LPPO modulates the proliferation and differentiation of keratinocytes as the principal component of the epidermis. The panel of studied keratins (K8, K14, and K19) and significant prevalence of E over N Cadherin revealed the normal status of keratinocytes in the absence/presence of LPPO. The ability of TGF-β1 either to induce epithelial-to-mesenchymal transition^34^ (E to N Cadherin switch) or to reduce proliferation^35^ was also not modulated by LPPO.

In the in vivo study we used a modified experimental model originally adapted to study antimicrobials for their ability to reduce *Acinetobacter baumannii*-induced wound infection in mice^36^. We demonstrated that this model is also suitable to study the clinically relevant *S. aureus* as wound healing impairing pathogen and NANO-LPPO as an efficient antibacterial wound dressing. LPPO exerted topical antibacterial activity when loaded at 5 and 10% with negligible systemic levels in liver and/or plasma. Similarly, a bioactive antibacterial (with sustained amoxicillin release for up to 1 week) bilayer PCL/gelatin nanofibrous scaffold hindered bacterial growth and promoted full-thickness wound healing^37^. Moreover, our newly developed dressing also slightly improved granulation tissue formation and this effect persisted when NANO was loaded with LPPO. The healing promoting effect can even be improved by specific modifications of the material structure, for example by combining the PCL with fish collagen and covalently bonded chitooligosaccharides^38^. Another scaffold comprised two supportive PCL-chitosan layers (accelerating healing) on the sides and a polyvinyl alcohol-metformin hydrochloride (down-regulating expression of fibrosis-related genes) in the middle^39^. From this point of view fabricated scaffolds are promising candidates for treatment (reducing fibrosis/infection and facilitating repair/regeneration) of full-thickness wounds in vivo.

## Conclusion

In the present study, we have demonstrated that the PCL-based/LPPO-loaded active wound dressing (NANO-LPPO) is effective in preventing *S. aureus*-induced wound infection in mice. The in vitro cytotoxicity and morphology assays indicated that the PCL-LPPO nanofibrous material has promising biocompatibility. LPPO is likely released by two main mechanisms, i.e. diffusion from the PCL and degradation of the PCL. LPPO exerted topical antibacterial activity when loaded at 5 and 10%. The most interesting and novel feature of the antibacterial dressing represents the ability of bacteria to increase the LPPO release rate into the wound. In detail, the enzymatic degradation of the nanofibrous scaffold is promoted by LPPO itself and also by (microbial) lipases. In other words the more bacterial burden in the wound will be present the more antibacterial compound will be released. A mechanism that may be found clinically relevant also for other medical applications (e.g. suture material). In summary, PCL-LPPO represents a promising strategy to replace traditional wound dressings with a potential to promote wound repair also in clinical settings. Nevertheless, the use of a murine model represents a limitation of our study due to interspecies variability. Further pre-clinical studies conducted on large animal models physiologically/evolutionary closer to humans are thus warranted by the presented set of data.

## Materials and methods

### LPPO

The second generation LPPO DR-6180 (Fig. S4) was synthesized in multigram quantities according to the procedure reported previously^6^.

### Nanofibrous materials preparation and loading with LPPO

Nanofibrous wound dressings were prepared by blending electrospinning technology^18,40^. Four nanofibrous (polycaprolactone—PCL) materials with different concentrations of LPPO were prepared (0, 2, 5, and 10 wt%). All concentrations were calculated for dry matter (excluded solvent). Firstly, LPPO was dissolved in chloroform/ethanol (Penta s.r.o., Praha, Czech Republic) solvent mixture at a weight ratio of 8:2 (solution for preparation of NANO supplemented with LPPO 2% was prepared by dissolving 0.32 g of LPPO in 84 g of solvent mixture; solution for preparation of PCL supplemented with 5% LPPO was prepared by dissolving 0.8 g of LPPO in 84 g of solvent mixture; solution for preparation of PCL supplemented with 10% LPPO was prepared by dissolving 1.6 g of LPPO in 84 g of solvent mixture). The ultrasound (40 kHz, 50 W) was then applied for 30 min to enhance the solubilization of LPPOs and ensure homogeneity of the spinning solutions using an ultrasonic bath (Shesto, Watford, UK). Then 16 g of polycaprolactone (Merck, Praha, Czech Republic) with an average molecular weight M_n_ 45,000 was dissolved in each solution. The polymer concentration used for obtaining continuous fibrous meshes was 16 wt% (LPPO is not included in this ratio). All materials were electrospun using Nanospider NS 1WS500U (Elmarco a.s., Liberec, Czech Republic) on polypropylene spunbond microfiber nonwoven (Pegatex S; 20 g/m^2^; fiber diameter 20 µm; PFNonwovens Czech, Czech Republic). For electrospinning, a positive voltage was applied to the wire (i.e., the spinning electrode) and a negative voltage was applied to the collector; the potential difference was 40 kV. The distance between the spinning electrode and the collector was 160 mm. The temperature during the experiments was 22 ± 5 °C, with a relative humidity of 40 ± 5%. Produced samples were finally sterilized in the flow box using a UV light for 10 min for each side.

### Scanning electron microscopy (SEM) and morphological analysis of PCL

Morphological analysis was performed by scanning electron microscopy (Vega Tescan 3, Tescan, Brno, Czech Republic) after 7 nm of gold sputter coating (Quorum Q50ES, Quorum Technologies, Laughton, Great Britain). Samples were analyzed at an accelerating voltage of 10 kV. The average fiber diameter and the diameter distribution were evaluated using NIS Elements (Laboratory imaging s.r.o., Prague, Czech Republic) using 300 randomly selected and measured fields per material. Histograms were used to summarize data from fiber diameter/distribution measurement.

### Degradation analysis of PCL-LPPO materials and measurement of LPPO release kinetics

Samples (n = 3) were cut from prepared electrospun planar materials to final weight of 50 ± 0.05 mg. Individual samples were placed in 5 mL tubes with 5 mL of degradation solution composed of phosphate buffered saline (PBS) buffer (pH = 7.4), 0.02% sodium azide and either lipase (Lipase from *Pseudomonas cepacia*, Merck, Darmstadt, Germany) in a concentration of 5 U/mL or without lipase (Table 1). The enzymatic degradation of the samples was performed at 37 °C for 5 days, without shaking. Samples of the solution were taken for subsequent HPLC analysis after 1, 2, 4, 8, and 12 h during the first day and then every 24 h. The buffer/enzyme solution was replaced at the same time periods, thus the enzyme activity remained constant throughout the entire experiment. Samples of solutions were analyzed for the LPPO content by HPLC (see below). Remaining nanomaterial samples were then washed in distilled water and dried at room temperature and further analyzed for weight loss and morphology by scanning electron microscopy (SEM).

The HPLC analyses were performed using Dionex Ultimate 3000 HPLC system equipped with a DAD 3000 UV–VIS diode array detector (Dionex, Sunnyvale, California, USA) controlled by Chromeleon 6.80 SR12 software on Phenomenex Kinetex Hilic column (particle size 2.6 µm, column dimensions 150 mm × 4.6 mm) at 40 °C, at a flow rate of 1.4 mL/min. Samples were diluted 1/4 with pure acetonitrile, vortexed for approximately 30 s, and consecutively filtered (nylon syringe filters, 0.22 µm, Chromservis, Prague, Czech Republic). The injection volume was 20 µL. Wavelengths of 200, 207, 262 and 280 nm were used for the detection of LPPO. The gradient elution program is shown in Table S4. LPPO was quantified by comparison with external standards of LPPO DR-6180. The calibration levels used were 50, 100, 200, 500 and 1000 mg/L.

### Wettability of PCL

The wettability of PCL electrospun nanofibrous materials (supplemented with or without LPPO) was tested by microtensiometer Krüss K121 (Krüss GmbH, Germany). Dynamic wetting was tested by the Washburn adsorption test. Similar surface densities of these samples were used: (1) PCL (NANO) and density of 27.1 ± 1.3 gm^2^ and (2) PCL supplemented with 10 wt% of LPPO (NANO-LPPO10%) and density of 28.3 ± 1.8 gm^2^. The sample of 30 mm width and 40 mm length was cut and placed into the holder for foils. The holder with nanofibrous sample was then inserted into the clip, with the bottom sample edge arranged in a horizontal position and parallel orientation of the sample edge during immersion in the test liquid (HPLC water, VWR Int., Stříbrná Skalice, Czech Republic). The ambient conditions were temperature of 22 °C and 58% relative humidity. The wicking, weight of water wicked into the fibrous system in time, into NANO and NANO-LPPO10% was measured five times for each condition. The spontaneous wicking of porous materials even of textiles or nanofibrous layers can be described by the Washburn equation^20^. The results were expressed in the form of average squared mass gain over time at the beginning of wicking represented by the direction of the regression curve of the wicking rate (Fig. S1).

### In vitro study in human dermal fibroblasts (HDF) and human keratinocyte cell line (HaCaT)

HDFs were isolated from skin specimens, as previously described^41^, obtained from two healthy donors during aesthetic surgery with the informed consent of patients (in complete agreement with the Helsinki Declaration after approval by the Ethical Committee of the University Hospital Královské Vinohrady) at the Department of Aesthetic Surgery (Third Faculty of Medicine, Charles University, Prague, Czech Republic). HDF cultures were expanded in Dulbecco's modified Eagle medium (DMEM) supplemented with 10% fetal bovine serum (FBS; Biochrom, Berlin, Germany) and penicillin/streptomycin (Biochrom). They were further culturing at 37 °C and 5% CO_2_. Cells at passages 7–8 were used in all experiments.

The HaCaT cell line^42^, used in the present study was obtained from Cell Lines Service (Eppelheim, Germany). Cells were cultured in Dulbecco’s Modified Eagles Medium (DMEM) supplemented with 10% fetal bovine serum (FBS) and antibiotics (streptomycin and penicillin) (all from Biochrom, Berlin, Germany).

In all in vitro experiments, TGF-β1 (PeproTech, London, UK) at 30 ng/mL^43^ was used as a positive control to evaluate the ability of HDFs to undergo myofibroblast differentiation. We examined whether LPPO modulates TGF-β1 signaling (both canonical and non-canonical). In parallel, LPPO and/or TGF-β1 effect was also studied in HaCaT cells. A standard cultivation medium was used as control.

### MTS assay

Cell viability and proliferation were determined using colorimetric microculture assay with MTS (3-(4,5-dimethylthiazol-2-yl)-5-(3-carboxymethoxyphenyl)-2-(4-sulfophenyl)-2*H*-tetrazolium) dye (Promega, Madison, WI, USA)^44^. Cells were seeded (HDFs—3000 cells/well; HaCaT—10,000 cells/well) into 96-well-plates in culture medium (10% FBS). After 24 h the medium was replaced with a medium containing LPPO (0.1, 1, 5, 10, 25, 50, and 100 mg/L) in the presence or absence of TGF-β1 (PeproTech). Following the next 72 h MTS (at a final concentration of 0.21 mg/mL) was added into the culture medium. After an additional 3 h, cell proliferation was evaluated by measuring the absorbance at wavelength 490 nm using the automated Cytation™ 3 Cell Imaging Multi-Mode Reader (Biotek, Winooski, VT, USA). The absorbance of control wells was taken as 100%, and the results were expressed as a percentage of the untreated control. The experiments were performed in technical triplicates and repeated three times.

### Migration (wound healing) assay

The migration of HaCaT cells was evaluated using a scratch assay. Briefly, a confluent layer of cells cultured on a 6-well plate was scratched using a pipette tip creating a “wound”. Afterwards, the medium was replaced with a medium containing LPPO at 1, 5 and 10 mg/L in the presence or absence of TGF-β1 (PeproTech). The wounded area was photographed at the beginning (0 h) and in 24 h. The migration distance (gap area) was determined using NIS Elements (Nikon, Tokyo, Japan) and expressed as a percentage of the original gap created at 0 h. The experiments were performed in technical duplicates and repeated twice.

### Western blot analysis

Based on the MTS assay we selected only non-toxic concentrations (1, 5, 10 mg/L) of LPPO for further western blot analysis. HDFs and HaCaTs were seeded on Petri dishes at the density of 5000 and 10,000 cells/cm^2^, respectively and cultivated for 7 days with LPPO in the presence or absence of TGF-β1. The set of primary and secondary antibodies applied in the analysis is shown in Table S5. The analysis was performed as described previously^45^. Briefly, cells were washed with cold PBS and collected in Laemmli sample buffer (100 mM TRIS–HCL pH approx. 6.8, 10% glycerol, 2% SDS) containing protease and phosphatase inhibitors (Sigma-Aldrich, St. Louis, MO, USA). Immediately after collection, cells were disrupted using a sonicator (QSonica, 40% amplitude, 15 s). After boiling (95 °C, 5 min), samples were separated in SDS-PAGE gel (10% Bis–Tris) and transferred to PVDF membrane using iBlot 2 (Thermo Fischer Scientific). Following 1 h of blocking in 5% NFDM/BSA (non-fat dry milk/bovine serum albumin) dissolved in TBS (tris-buffered saline) with 0.1% Tween at room temperature, membranes were incubated with the primary antibody at 4 °C overnight. The next day, the membranes were washed in TBS-Tween (3 × 5 min) and incubated with HRP-conjugated secondary antibodies for 1 h at room temperature. After incubation, the membranes were again washed in TBS-Tween (3 × 5 min), and protein bands were detected using ECL (SuperSignal West Pico PLUS chemiluminescent Substrate, Thermo Fischer Scientific) and the signal was acquired at MF-ChemiBis 2.0 (DNR Bio-Imaging Systems). β-actin was used as a sample loading control. The chemiluminescent signal of all detected proteins was quantified using the Image Studio (LI-COR) western blot densitometry software and normalized to β-actin.

### Imunofluorescence of cultured cells

HDFs and HaCaTs were seeded at a density of 5000 and 10,000 cells/cm^2^, respectively. Both cells were cultivated with LPPO (1, 5 and 10 mg/L) in the presence or absence of TGF-β1 (PeproTech) for 7 days. The set of primary and secondary antibodies applied in the analysis is shown in Table S6. The analysis was performed as described previously^46^. Briefly, tested specimens were fixed with 2% buffered paraformaldehyde (pH = 7.2) for 5 min. and washed with PBS. Cells were permeabilized by exposure to Triton X-100 (Sigma-Aldrich), and sites for antigen-independent binding of antibodies were blocked by incubation with porcine serum albumin. Commercial antibodies were used at concentrations recommended by the suppliers. All specimens were mounted to Vectashield (Vector Laboratories, Eching, Germany) and inspected by using an Eclipse 90i microscope equipped with filter blocks for the three types of dyes (Nikon, Tokyo, Japan) as well as a Hamamatsu CCD camera (Hamamatsu, Shizuoka, Japan) and a computer-assisted image analysis system NIS (Nikon, Tokyo, Japan).

### Animal study

The experimental conditions complied with the European rules of animal care and welfare in compliance with the ARRIVE guidelines. The experiment was approved by the Ministry of Health of the Czech Republic (MZDR 20378/201/-4/OVZ).

### Mice

Inbred male Balb/c (Balb) (Charles River Laboratories, Munich, Germany) mice, 8–12 weeks of age weighing 17–22 g, were used in the experiment. Breading of mice and all experimental procedures were conducted under specific-pathogen-free (SPF) conditions in an SPF Animal Facility, National Institute of Public Health, Prague, Czech Republic. Animals were housed under 12 h day/night light cycle and standard environmental conditions (22 °C, 55% relative humidity). Mice had free access to sterile water and commercial ST1 diet (Velaz, Prague, Czech Republic) ad libitum. Prior to the wound healing experiment all mice were housed in groups of three, in sanitized cages on sterile paper bedding, and were provided with environmental enrichment, including in-cage plastic housing.

The allocation of mice (n = 54) in treatment groups is shown in Table 2. All mice were sacrificed under general anesthesia by cervical dislocation 7 days after surgery. Wound samples were removed for histological, bacteriological culture-based and bacteriological quantitative PCR-based evaluations (see below). In addition, plasma and liver were also examined for the presence of LPPO residues.

**Table 2:** Allocation of mice (n = 54) in treatment groups.

Group (treatment/infection)	Control	NANO	NANO-LPPO2%	NANO-LPPO5%	NANO-LPPO10%
None	n = 6 (S1–S6)	n = 6 (S7–S12)	n = 6 (S13–S18)	n = 6 (S19–S24)	n = 6 (S25–S30)
+ *S. aureus*		n = 6 (S31–S36)	n = 6 (S37–S42)	n = 6 (S43–S48)	n = 6 (S49–S54)

NANO: Polycaprolactone-based nanofiber/wound dressing; LPPO: lippophosphonoxin DR-6180.

### Wound model

Two days before wounding mice received 100 mg/kg of cyclophosphamide via intraperitoneal (i.p.) injection^36^. On day 0, the day of wounding and inoculation, mice were anesthetized with Isoflurane (Aerrane 100%, Baxter, Lessines, Belgium). Hair was clipped from the cervical to mid-lumbar dorsum, and the skin was scrubbed with iodine solution followed by an ethanol rinse. An 8-mm disposable skin biopsy punch (Kai Medical, Kai Europe, Solingen, Germany) was used to create a full-thickness skin defect overlying the thoracic spinal column and the adjacent musculature. Aliquots of 25 μL containing the inoculum (see below) in a PBS suspension were pipetted into the wound and allowed to absorb. A circular cutout (30 mm in diameter) of transparent dressing (TegadermRoll; 3 M Health Care, St. Paul, MN) was placed over the wound with or without PCL-scaffolds supplemented or not with LPPO at 2, 5, and 10% (NANO, NANO-LPPO2%, NANO-LPPO5%, and NANO-LPPO10%).

### Bacterium and inoculum preparation

*Staphylococcus aureus* strain CNCTC 5480 (= ATCC 29213) obtained from the Czech National Collection of Type Cultures (https://www.szu.cz/cnctc) was used in the present study. To prepare an inoculum for experimental wound infection, 15 mL of Luria–Bertani (LB) medium (Oxoid Ltd, Basingstoke, UK) in a 100-mL Erlenmeyer flask was inoculated with 50 μL of cell suspension with a density of ≈ 10^8^ colony-forming units [CFU] per mL, which was prepared in saline from an overnight CNCTC 5480 culture on a sheep blood agar (BA) plate (Oxoid). The inoculated medium was then cultured at 37 °C with shaking in a thermostatically controlled water bath. After 3.5 h, 1 mL of the mid-exponential growth phase culture was taken and the harvested cells were washed twice and finally diluted in PBS (pH = 7.2). The bacterial quantity was determined by the measurement of the optical density (OD) and based on the known correlation between OD and CFU values the final suspension was adjusted to contain ≈ 2.5 × 10^4^ CFU/mL of PBS. The bacterial density of the final suspension was checked by CFU counting using serial dilution and plating onto LB agar plates.

### Histology of open wounds

Skin-wound specimens were removed from sacrificed mice and routinely processed for histological staining (fixation in 4% buffered formaldehyde, dehydration using increasing concentrations of ethanol, paraffin embedding, sectioning, and staining). Deparaffinized Sections (2–3 µm thick) were stained with hematoxylin–eosin (H + E—basic staining), Sirius Red (SR—collagen staining) and Gram staining (to distinguish between gram-positive/negative bacteria).

### Culture-based characterization of experimental bacterial wound infection

Inoculated blood agar (BA) plates were cultured aerobically at 37 °C for 20 h. The resulting bacterial growth was visually evaluated and if positive, bacteria were isolated, cultured on BA and checked for their identity with *S. aureus* CNCTC 5480. Species identification of isolates was based on whole-cell profiling by matrix-assisted laser desorption/ionization time-of-flight (MALDI-TOF) MS using the Microflex LT instrument and BioTyper software version 3.1 (Bruker Daltonics, Bremen, Germany). Isolates identified as *S. aureus* were then checked for their identity with CNCTC 5480 at the strain level using SmaI-based macrorestriction analysis based on a previously published protocol^47^.

### Quantitative PCR-based characterization of experimental bacterial wound infection

#### Sample homogenization

Please see Supplementary material.

#### Isolation of chromosomal DNA

Please see Supplementary material.

#### qPCR

Please see Supplementary material^48,49^.

#### Blood sampling and preparation of liver tissue homogenates

Please see Supplementary material.

#### Plasma sample preparation

Please see Supplementary material.

#### Liver sample preparation

Please see Supplementary material.

#### Analysis of LPPO residues in mouse plasma and liver by LC–MS/MS

Please see Supplementary material.

#### HPLC/MS method

Please see Supplementary material.

#### Chromatographic and mass spectrometric conditions

Please see Supplementary material (Table S7).

#### Analytical method optimization

Please see Supplementary material.

### Statistical analysis

Data from the in vitro experiments (MTS-assay) are expressed as mean standard deviation and were compared by one-way analysis of variance followed by Dunnetts's post-hoc test. Significance was accepted at *p* < 0.05.

## Supplementary Information

Supplementary Information.
