# Supplementary material for: Novel lipophosphonoxin-loaded polycaprolactone electrospun nanofiber dressing reduces Staphylococcus aureus induced wound infection in mice
Source: Sci Rep. 2021 Sep 3;11:17688. doi: 10.1038/s41598-021-96980-7 (PMC8417216; doi:10.1038/s41598-021-96980-7)
Supplement: Supplementary file 1 — Supplementary Information. [file 41598_2021_96980_MOESM1_ESM.docx]

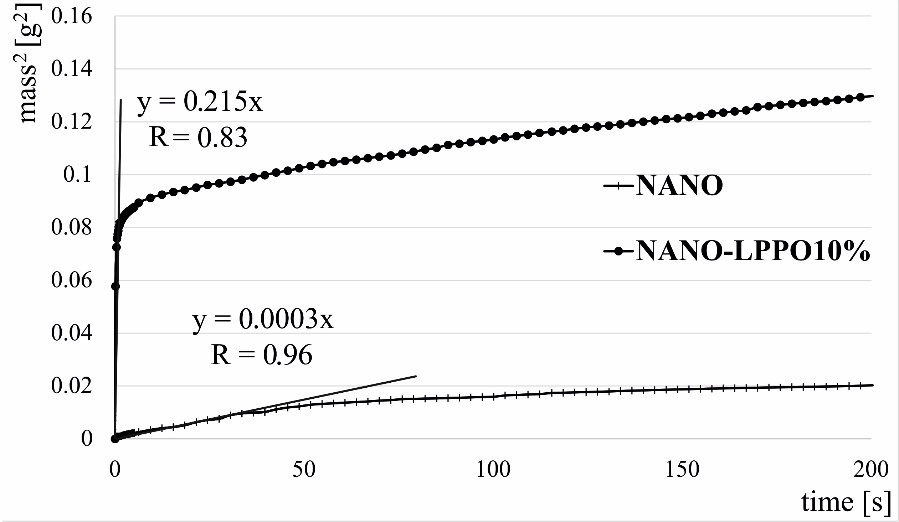


**Figure S1.** Average values of squared weight gain at the measurement up to 200 s for NANO (vertical short lines) and NANO-LPPO10% (black circles). The initial velocities are expressed by linear trend equation (linear regression curve) and regression coefficient R. The slope of the line is denoted as “a” in equation y=ax, thus the slope of the line represents the wicking rate.


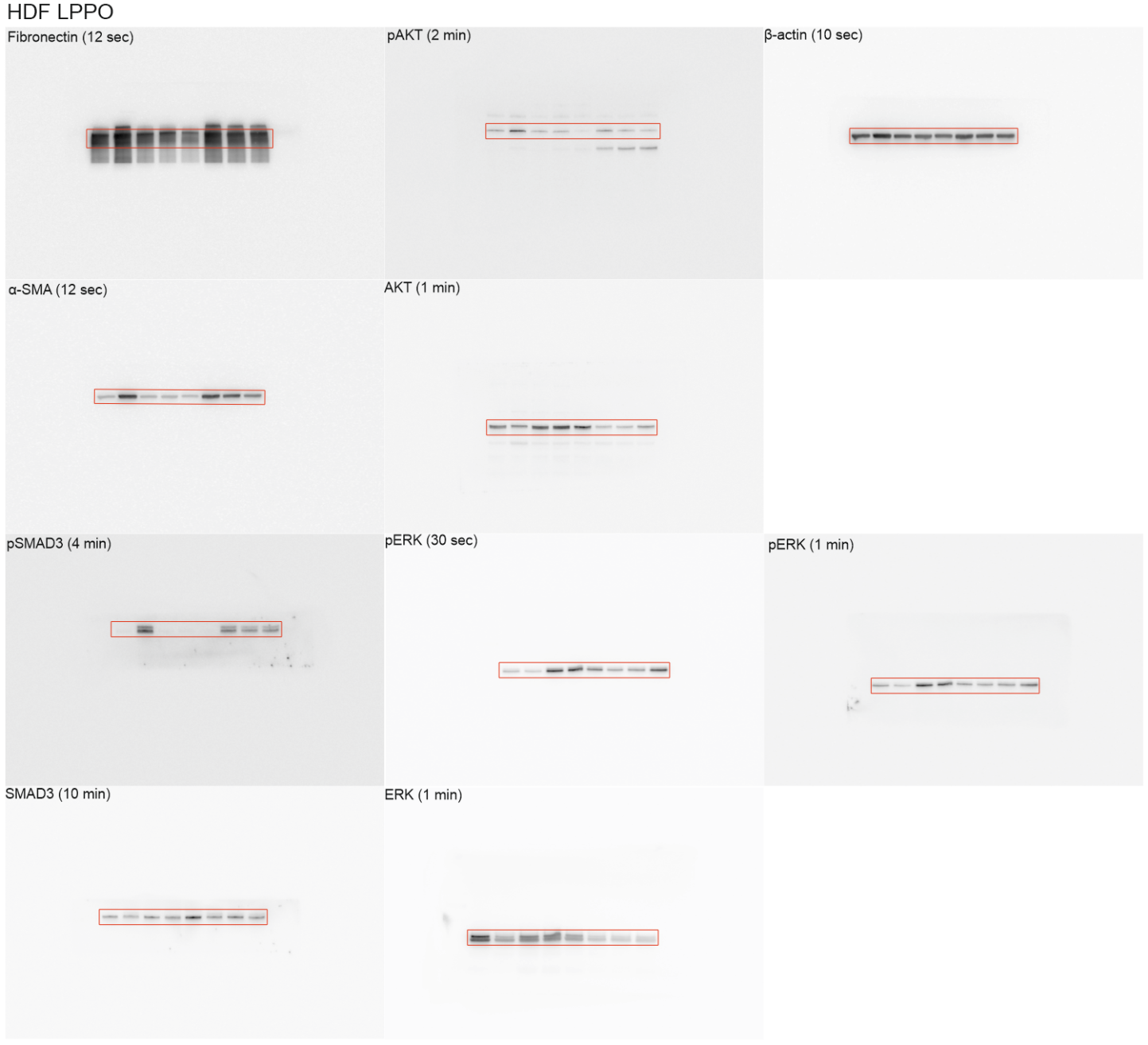


**Figure S2.** Full-length gel from the western blot analysis of studied proteins in HDFs.


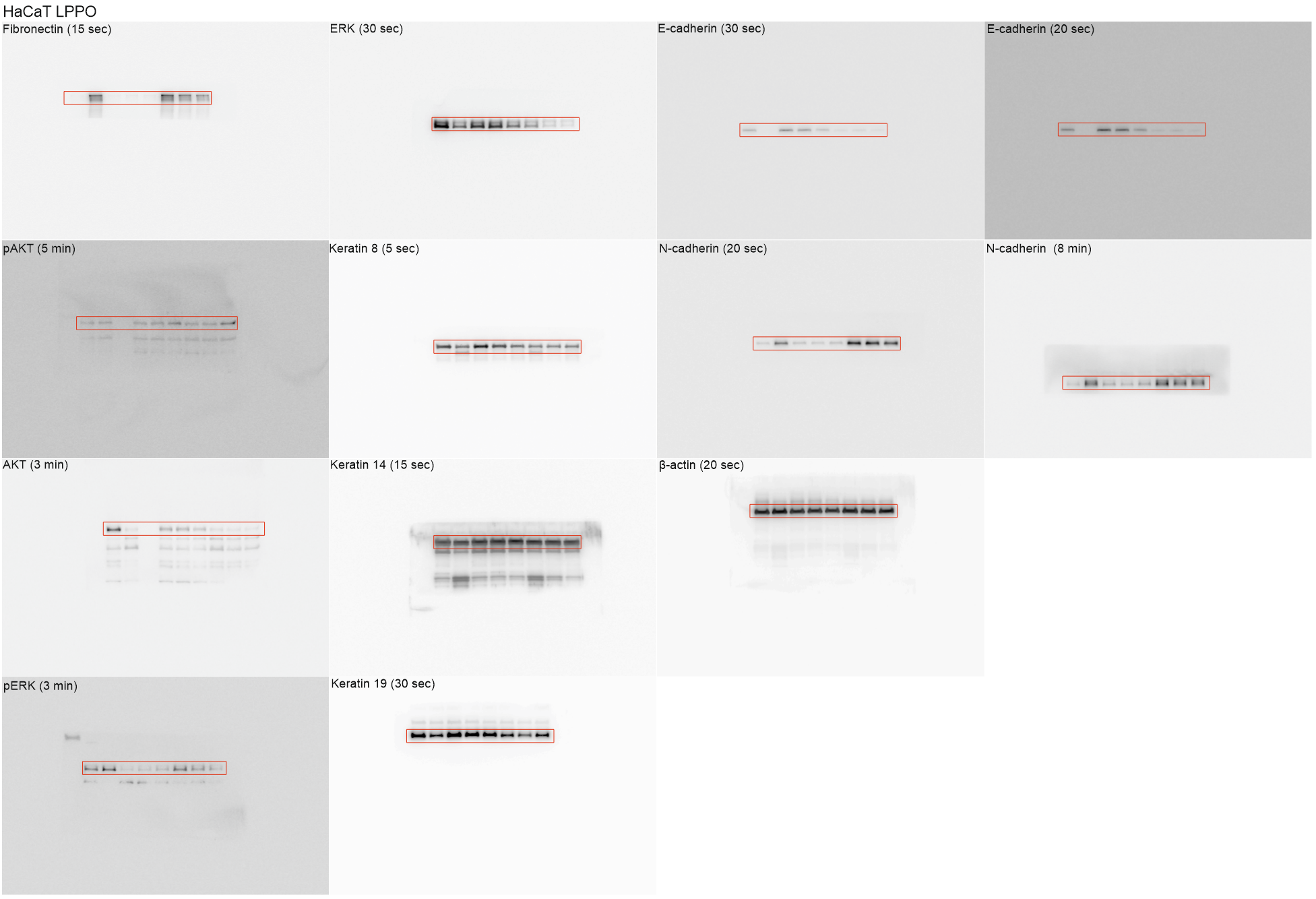


**Figure S3.** Full-length gel from the western blot analysis of studied proteins in HaCaTs.

**Table S1.** Grown cultures validated by MALDI-TOF.

| **Sample** | **Skin**  **(visual assessment)** | | **Wound**  **(visual assessment)** | | **Skin**  **(ident. MALDI-TOF)** | | | | **Wound**  **(ident. MALDI-TOF)** | | **Design** | | | |
| --- | --- | --- | --- | --- | --- | --- | --- | --- | --- | --- | --- | --- | --- | --- |
| **S1** | neg | | 1 col. hem- | |  | | | | S. sciuri | | Tegaderm | | | |
| **S2** | neg | | neg | |  | | | |  | | Tegaderm | | | |
| **S3** | neg | | neg | |  | | | |  | | Tegaderm | | | |
| **S4** | 1 col. hem- | | ≈5 col. hem- | | S. sciuri | | | | S. sciuri | | Tegaderm | | | |
| **S5** | 1 col. hem- | | 2 col. hem- | | S. sciuri | | | | S. sciuri | | Tegaderm | | | |
| **S6** | neg | | 1 col. hem- | |  | | | | S. sciuri | | Tegaderm | | | |
| **S7** | + hem- | | + hem- | | S. aureus hem- | | | | S. aureus hem- | | PCL+tegaderm | | | |
| **S8** | + hem- | | + hem- | | S. aureus hem- | | | | S. aureus hem- | | PCL+tegaderm | | | |
| **S9** | + hem- | | + hem- | | S. aureus hem- | | | | S. aureus hem- | | PCL+tegaderm | | | |
| **S10** | 5 col. hem- | | neg | | S. aureus hem- | | | |  | | PCL+tegaderm | | | |
| **S11** | neg | | neg | |  | | | |  | | PCL+tegaderm | | | |
| **S12** | + hem- | | + hem- | | E. gallinarum | | | | E. gallinarum | | PCL+tegaderm | | | |
| **S13** | neg | | neg | |  | | | |  | | PCL+tegaderm+LPPO 2% | | | |
| **S14** | neg | | neg | |  | | | |  | | PCL+tegaderm+LPPO 2% | | | |
| **S15** | neg | | neg | |  | | | |  | | PCL+tegaderm+LPPO 2% | | | |
| **S16** | neg | | 2 col. hem- | |  | | | | S. sciuri | | PCL+tegaderm+LPPO 2% | | | |
| **S17** | neg | | 4 col. hem- | |  | | | | S. sciuri | | PCL+tegaderm+LPPO 2% | | | |
| **S18** | 1 col. hem- | | neg | | S. sciuri | | | |  | | PCL+tegaderm+LPPO 2% | | | |
| **S19** | neg | | neg | |  | | | |  | | PCL+tegaderm+LPPO 5% | | | |
| **S20** | neg | | 1 col. hem- | |  | | | | S. sciuri | | PCL+tegaderm+LPPO 5% | | | |
| **S21** | neg | | neg | |  | | | |  | | PCL+tegaderm+LPPO 5% | | | |
| **S22** | neg | | neg | |  | | | |  | | PCL+tegaderm+LPPO 5% | | | |
| **S23** | 5 col. vir+ | | 3 col. vir+ | | Alfa-streptococci | | | | Alfa-streptococci | | PCL+tegaderm+LPPO 5% | | | |
| **S24** | neg | | neg | |  | | | |  | | PCL+tegaderm+LPPO 5% | | | |
| **S25** | neg | | neg | |  | | | |  | | PCL+tegaderm+LPPO 10% | | | |
| **S26** | neg | | neg | |  | | | |  | | PCL+tegaderm+LPPO 10% | | | |
| **S27** | neg | | neg | |  | | | |  | | PCL+tegaderm+LPPO 10% | | | |
| **S28** | neg | | neg | |  | | | |  | | PCL+tegaderm+LPPO 10% | | | |
| **S29** | 1 col. vir+ | | 1 col. vir+ | | Alfa-streptococci | | | | Alfa-streptococci | | PCL+tegaderm+LPPO 10% | | | |
| **S30** | neg | | neg | |  | | | |  | | PCL+tegaderm+LPPO 10% | | | |
| **S31** | **+ hem+** | | **+ hem+** | | **S. aureus** | | | | **S. aureus** | | PCL+tegaderm+**S. aureus** | | | |
| **S32** | **++ hem+** | | **++ hem+** | | **S. aureus** | | | | **S. aureus** | | PCL+tegaderm+**S. aureus** | | | |
| **S33** | **++ hem+** | | **++ hem+** | | **S. aureus** | | | | **S. aureus** | | PCL+tegaderm+**S. aureus** | | | |
| **S34** | **++ hem+** | | **++ hem+** | | **S. aureus** | | | | **S. aureus** | | PCL+tegaderm+**S. aureus** | | | |
| **S35** | **++ hem+** | | **++ hem+** | | **S. aureus** | | | | **S. aureus** | | PCL+tegaderm+**S. aureus** | | | |
| **S36** | **++ hem+** | | **++ hem+** | | **S. aureus** | | | | **S. aureus** | | PCL+tegaderm+**S. aureus** | | | |
| **S37** | **++ hem+** | | **++ hem+** | | **S. aureus** (+ S. aureus hem-) | | | | **S. aureus** | | PCL+tegaderm+LPPO 2%+**S. aureus** | | | |
| **S38** | **++ hem+** | | **++ hem+** | | **S. aureus** | | | | **S. aureus** | | PCL+tegaderm+LPPO 2%+**S. aureus** | | | |
| **S39** | **++ hem+** | | **++ hem+** | | **S. aureus** | | | | **S. aureus** | | PCL+tegaderm+LPPO 2%+**S. aureus** | | | |
| **S40** | 1 col. hem+ | | **++ hem+** | | **S. aureus** | | | | **S. aureus** | | PCL+tegaderm+LPPO 2%+**S. aureus** | | | |
| **S41** | **+ hem+** | | **+ hem+** | | **S. aureus** | | | | **S. aureus** | | PCL+tegaderm+LPPO 2%+**S. aureus** | | | |
| **S42** | 1 col. hem+ | | **++ hem+** | | **S. aureus** | | | | **S. aureus** | | PCL+tegaderm+LPPO 2%+**S. aureus** | | | |
| **S43** | neg | | 3 col. hem- | |  | | | | S. sciuri | | PCL+tegaderm+LPPO 5%+**S. aureus** | | | |
| **S44** | neg | | 1 col. hem- | |  | | | | S. sciuri | | PCL+tegaderm+LPPO 5%+**S. aureus** | | | |
| **S45** | 1 col. | | 4 col. hem- | | not identified | | | | S. sciuri | | PCL+tegaderm+LPPO 5%+**S. aureus** | | | |
| **S46** | neg | | neg | |  | | | |  | | PCL+tegaderm+LPPO 5%+**S. aureus** | | | |
| **S47** | neg | | neg | |  | | | |  | | PCL+tegaderm+LPPO 5%+**S. aureus** | | | |
| **S48** | **+ hem+ (≈10 col.)** | | **++ hem+** | | **S. aureus** (+ S. aureus hem-) | | | | **S. aureus** | | PCL+tegaderm+LPPO 5%+**S. aureus** | | | |
| **S49** | 3 col. vir+ | | neg | | NI (alfa-streptococci?) | | | |  | | PCL+tegaderm+LPPO 10%+**S. aureus** | | | |
| **S50** | neg | | 3 col. hem- | |  | | | | S. sciuri | | PCL+tegaderm+LPPO 10%+**S. aureus** | | | |
| **S51** | neg | | neg | |  | | | |  | | PCL+tegaderm+LPPO 10%+**S. aureus** | | | |
| **S52** | 4 col. vir+ | | neg | | NI (alfa-streptococci?) | | | |  | | PCL+tegaderm+LPPO 10%+**S. aureus** | | | |
| **S53** | 2 col. vir+ | | neg | | NI (alfa-streptococci?) | | | |  | | PCL+tegaderm+LPPO 10%+**S. aureus** | | | |
| **S54** | 2 col. vir+ | | neg | | Alfa-streptococci | | | |  | | PCL+tegaderm+LPPO 10%+**S. aureus** | | | |
| +, ++ |  | | |  | |  | | | |  | |  |  |  |
| col. |  |  | | | | |  |  | | | | |  |  |
| cont. |  | | |  | |  | | | |  | |  |  |  |
| hem+ |  | | |  | |  | | | |  | |  |  |  |
| hem- |  | | |  | |  | | | |  | |  |  |  |
| neg |  | |  | |  | | | |  | |  | | | |
| vir+ |  | | |  | |  | | | |  | |  |  |  |

**Table S2.** Concentration of LPPO in the plasma.

| Sample no. | C (ng/mL) | C (nmol/L) | Group description | Group mean (nmol/L) | RSD (%) |
| --- | --- | --- | --- | --- | --- |
| S13-S18 | 1.62 | 2.14 | T+NANO-LPPO2% | 2.46 | 27 |
| S19-S24 | 3.96 | 5.21 | T+NANO-LPPO5% | 4.64 | 18 |
| S25-S30 | 9.56 | 12.59 | T+NANO-LPPO10% | 14.51 | 15 |
| S31-S36 | 0.00 | 0.00 | T+NANO+*S.a.* | 0.00 |  |
| S37-S42 | 3.39 | 4.47 | T+NANO-LPPO2%+*S.a.* | 4.17 | 43 |
| S43-S48 | 1.58 | 2.08 | T+NANO-LPPO5%+*S.a.* | 4.31 | 41 |
| S49-S54 | 8.49 | 11.18 | T+NANO-LPPO10%+*S.a.* | 12.07 | 31 |

T - Tegaderm; NANO - polycaprolactone-based nanofiber wound dressing; LPPO - lippophosphonoxin; *S.a.* - Staphylococus aureus

**Table S3.** Concentration of LPPO in the liver.

| Sample no. | C (ng/mL) | C (nmol/L) | C (nmol/g) | Group description | Group mean (nmol/L) | RSD (%) |
| --- | --- | --- | --- | --- | --- | --- |
| S13-S15 | 113.8 | 149.8 | 0.899 | T+NANO-LPPO2% | 0.744 | 29 |
| S19-S21 | 222.5 | 292.9 | 1.758 | T+NANO-LPPO5% | 1.298 | 33 |
| S25-S27 | 659.2 | 868.0 | 5.208 | T+NANO-LPPO10% | 4.634 | 33 |
| S31-S32 | 0.00 | 0.00 | 0.00 | T+NANO+*S.a.* | 0.00 |  |
| S37-S39 | 72.23 | 95.11 | 0.571 | T+NANO-LPPO2%+*S.a.* | 0.522 | 26 |
| S43-S45 | 127.9 | 168.4 | 1.011 | T+NANO-LPPO5%+*S.a.* | 1.044 | 25 |
| S49-S51 | 161.7 | 212.9 | 1.277 | T+10%NANO-LPPO +*S.a.* | 1.385 | 98 |

T - Tegaderm; NANO - polycaprolactone-based nanofiber wound dressing; LPPO - lipophosphonoxin; *S.a.* - *Staphylococus aureus*

|  | *Staphylococcus aureus* MRSA 4591 | *Staphylococcus haemolyticus* 16568 |
| --- | --- | --- |
| **MIC (mg/L)** | 3.125 | 1.56 |

Seydlová G et al. Lipophosphonoxins II: Design, Synthesis, and Properties of Novel Broad Spectrum Antibacterial Agents. J Med Chem. 2017 Jul 27;60(14):6098-6118.

**Figure S4.** Synthesis of LPPO DR-6180 and MIC against *S. aureus*.

**Table S4.** Gradient program used in the HPLC analysis of LPPO release.

| Time (min) | A (%) | B (%) | C (%) |
| --- | --- | --- | --- |
|  | Acetonitrile | 5 mM formic acid in 5% ACN | 50 mM ammonium acetate in 75% ACN |
| 0.0 | 60 | 25 | 15 |
| 8.0 | 60 | 25 | 15 |
| 10.0 | 30 | 60 | 10 |
| 11.0 | 30 | 60 | 10 |
| 13.0 | 60 | 25 | 15 |
| 15.5 | 60 | 25 | 15 |

LPPO - lippophosphonoxin DR-6180

**Table S5.** Primary and secondary antibodies used for western blot.

| **Primary Antibody** | **Abbreviation** | **Host** | **Isotype** | **Clonality** | **Produced by** |
| --- | --- | --- | --- | --- | --- |
| α-smooth muscle actin | SMA | rabbit | IgG | monoclonal | CST,USA |
| Fibronectin | Fibr | rabbit | IgG | monoclonal | Abcam,UK |
| Phospho-ERK1/2 | pERK | rabbit |  | polyclonal | CST,USA |
| Phospho-AKT | pAKT | rabbit | IgG | monoclonal | CST,USA |
| Phospho-Smad3 | pSmad3 | rabbit | IgG | monoclonal | Abcam,UK |
| ERK1/2 | ERK | rabbit | IgG | monoclonal | CST,USA |
| Smad3 | Smad3 | rabbit |  | monoclonal | CST,USA |
| AKT | AKT | rabbit |  | polyclonal | CST,USA |
| β-actin | β-actin | rabbit | IgG | monoclonal | CST,USA |
| N-cadherin | N-cadherin | rabbit | IgG | polyclonal | ThermoFisher Scientific, USA |
| CD324(E-cadherin) | E-cadherin | rabbit | IgG1 | monoclonal | ThermoFisher Scientific, USA |
| Cytokeratin 14 | Keratin 14 | rabbit | IgG | polyclonal | ThermoFisher Scientific, USA |
| Cytokeratin 19 | Keratin 19 | mouse | IgG2a | monoclonal | ThermoFisher Scientific, USA |
| Cytokeratin 8 | Keratin t8 | mouse | IgG1 | monoclonal | ThermoFisher Scientific, USA |
| **Secondary Antibody** | **Abbreviation** | **Host** | **Isotype** | **Clonal** | **Produced by** |
| Anti-rabbit, HRP-linked |  | goat | IgG |  | CST,USA |
| Anti-mouse, HRP-linked |  | horse | IgG |  | CST,USA |

| **Table S6.** Primary and secondary antibodies used for immunofluorescence. | | | | | |  | |  |
| --- | --- | --- | --- | --- | --- | --- | --- | --- |
| **Primary Antibody** | **Abbreviation** | **Host** | **Produced by** | **Secondary Antibody** | **Produced by** | | **Channel** | |
| α-smooth muscle actin | SMA | Mouse monoclonal | DakoCytomation, Glostrup, Denmark | Goat anti-mouse | Sigma-Aldrich, St. Louis, MO, USA | | TRITC-red | |
| Fibronectin | Fibronectin | rabbit polyclonal | DakoCytomation, Glostrup, Denmark | Goat anti-rabbit | Sigma-Aldrich, St. Louis, MO, USA | | FITC-green | |
| Cytokeratin 14 | Keratin 14 | Rabbit polyclonal | ThermoFisher Scientific, USA | Goat anti-rabbit | Sigma-Aldrich, St. Louis, MO, USA | | FITC-green | |
| Cytokeratin 19 | Keratin 19 | Mouse monoclonal | ThermoFisher Scientific, USA | Goat anti-mouse | Sigma-Aldrich, St. Louis, MO, USA | | TRITC-red | |
| Cytokeratin 8 | Keratin 8 | Mouse monoclonal | ThermoFisher Scientific, USA | Goat anti-mouse | Sigma-Aldrich, St. Louis, MO, USA | | TRITC-red | |

*Quantitative PCR-based characterization of experimental bacterial wound infection*

*Sample homogenization*

Wound and skin mice tissue samples were divided into two groups – bacteria-free samples and samples with *S. aureus* to avoid cross-contamination. PBS (1 mL) was then added to each sample. Tissue disintegration was performed with Heidolph RZR2020 homogenizer (Heidolph Instruments GmbH & CO. KG, Schwabach, Germany) using a sterile PTFE pestle with stainless steel shaft and a borosilicate glass tube for each sample. Immediately after disintegration, samples were transferred into sterile 1.5 mL Eppendorf tubes and used for DNA isolation.

*Isolation of chromosomal DNA*

Chromosomal DNA (chDNA) was isolated using the High Pure Template Preparation Kit (Roche Diagnostic, Mannheim, Germany) according to the kit protocol. The only difference from the manufacturer's protocol was a prolongation of the lysozyme treatment from 15 to 60 min. Isolated chDNA was used for quantitative PCR (qPCR).

*qPCR*

Quantification of *S. aureus* in mice tissues was performed by qPCR using the Light Cycler LC480 (Roche Diagnostic, Mannheim, Germany). Separate 384 well plates were used for each sample group. 2 µL of each sample, in duplicates were spotted in the plate well together with 2.5 µL of Sybr Green Master Mix (Roche Diagnostic, Mannheim, Germany) and 0.5 µL of respective primers (total volume of 5 µL). PCR Temperature conditons: 1x 95 °C for 7 min followed by 45x[95 °C for 20 sec, 61 °C for 20 sec and 72 °C (in the single acquisition mode) for 35 sec]; this was followed by 1 melting curve cycle at 95 °C for 15 s, 55 °C for 1 min 1 s, and 95 °C (in the continuous acquisition mode with 10 acquisitions / °C) and 37 °C for 1 min 1 s. For *S. aureus* we used primers LK2822 (5'-GCGATTGATGGTGATACGGTI-3') and LK2823 (5'-AGCCAAGCCTTGACGAACTAAAGC-3') as described previously [1]. To quantitate the bacteria, we used standard curves with known, increasing amounts of DNA purified from *S. aureus*. The ΔCt method was used to determine the relative quantities of cDNAs [2]. The absolute quantities were then calculated from sample Ct values compared to the values obtained from the standard curves. Of note, the primers used in this study do not differentiate between various *S. aureus* strains. Nevertheless, the load of the strain used to infect the wound was high and the non-infected controls revealed *S. aureus* levels below the limit of detection.

*Blood sampling and preparation of liver tissue homogenates*

On day 7 (immediately prior to euthanasia) blood was collected in general anesthesia from the retro-orbital venous plexus with a capillary tube into EDTA Vacutainers. Plasma was separated by centrifugation and stored at –20 °C until further analysis.

Following euthanasia (by cervical dislocation), the liver was excised and immediately transferred into pre-cooled sterile containers and kept at 4 °C until homogenization. Consecutively, 6 mL of 0.5% solution of formic acid in acetonitrile/water (1:1) per gram of tissue was added to each container. Consecutively, electric motor driven Potter-Elvehjam Teflon/glass homogenizer (1200 RPM; HEi-Torque Value 100, Heidolph, Germany) was used to sample homogenization.

*Plasma*

Fifty μl of plasma was pipetted to the polypropylene tube and 6 μL of an internal standard solution containing 0.732 ng/μL of IS was added. This corresponds to an internal standard concentration of 87.84 ng/ml in the plasma sample. One hundred μl acetonitrile containing 0.5% formic acid was added, the tube was then vortex-mixed for 15 s at 2000 rpm and centrifuged for 2 min at 4000 rpm. The supernatant was transferred to a 0.3 mL polypropylene autosampler vial. Finally, 10 μL were injected into the chromatographic system.

*Liver*

The sample tube containing tissue and 6 mL of extraction solution 0.5% formic acid in acetonitrile/water binary mixture (1:1) was mixed well and centrifuged for 4 min at 4000 rpm. The supernatant (200 μL) was pipette to a polypropylene tube and mixed with 30 μL of internal standard solution at the concentration of 0.732 ng/μL to reach the final concentration of the internal standard at 109.8 ng/mL. The tube was vortex-mixed for 15 s at 2000 rpm and the supernatant was transferred to a 0.3 mL polypropylene autosampler vial. Finally, 5 μL of the sample was injected into the chromatographic system.

*Analysis of LPPO residues in mouse plasma and liver by LC-MS/MS*

Chemicals and Equipment: Test articles DR-6180 and DR-6328 (internal standard, IS) were supplied by the Institute of Organic Chemistry and Biochemistry AS CR (Prague, Czech Republic). Methanol (HPLC Super Gradient grade) was a product of Macron, Gliwice, Poland, acetonitrile (Ultra Gradient HPLC Grade) was manufactured by J.T. Baker, Phillipsburg, New Jersey, USA. Formic acid (98–-100%, puriss. p.a.) was delivered by Sigma-Aldrich (Prague, Czech Republic).

*HPLC/MS*

The system consisted of the P4000 pump, SCM 1000 degasser, AS3000 autosampler, TSQ Quantum Discovery Max triple quadrupole mass spectrometer with an electro-spray ion source and data station with Xcalibur software, version 2.0.7 (Thermo Electron Corporation, Waltham, MA, USA). The separation was performed on a reversed-phase column (Discovery Bio Wide Pore C5 20x4 mm, particle size 3 μm, Merck).

Preparation of Standard Solutions Stock solutions of DR-6180 and IS were made by dissolving the substance in methanol. As DR-6180 was supplied as a trihydrochloride salt, the calculated concentrations of DR-6180 were multiplied by a factor of 0.9119 to obtain concentrations of the free base. The diluted solutions were prepared by dilution of the stock solution with acetonitrile-1% formic acid (1:1). The solutions were stored in the darkness at –18 °C.

The working solution of internal standard (0.732 ng/μL) was prepared by the dilution of the stock solution with acetonitrile-1% aq. formic acid (1:1). The internal standard working solution must be the same within the whole analytical batch.

Preparation of Calibrators

The stock solution of the analyte was added to drug-free plasma in a volume not exceeding 5% of the plasma volume to prepare a calibration plasma sample with a concentration of 100.2 ng/mL. Less concentrated calibrators (1.236, 3.709, 11.13 and 33.38 ng/mL) were prepared by consecutive dilutions of the 100.2 ng/mL calibrator with blank plasma. The QC samples were prepared in a similar way. Two QC levels were prepared at 80.32 ng/ml (high level) and 4.725 ng/mL (low level). Similarly, blank tissue extract was spiked with a stock solution of the analyte to prepare a calibration sample of tissue extract with a concentration 676.0 ng/mL. Less concentrated calibrators (3.740, 13.71, 50.28 and 184.4 ng/mL) were prepared by consecutive dilutions of the 676.0 ng/mL calibrator with blank tissue extract. The QC samples were prepared in a similar way. Two QC levels were prepared at 386.3 ng/ml (high level) and 18.40 ng/mL (low level). The plasma and liver samples were stored in the freezer at -18 °C. The thawing was allowed in tepid water before processing the sample.

*Chromatographic and Mass Spectrometric Conditions*

The mobile phase consisted of methanol and 0.1% formic acid. The flow-rate was 0.4 mL/min at 35 °C. The gradient elution program shown in Table S7. was applied:

**Table S7.** Gradient program.

| Time (min) | Methanol (%) | 0.1% formic acid (%) |
| --- | --- | --- |
| 0.0 | 10 | 90 |
| 1.0 | 80 | 20 |
| 3.5 | 95 | 5 |
| 3.6 | 100 | 0 |
| 3.8 | 100 | 0 |
| 3.9 | 10 | 90 |
| 8.5 | 10 | 90 |

The volume of the gradient mixer was about 1.5 mL, consequently the gradient was delayed by about 3.8 min. Ionization of the analytes was carried out using electrospray ionization technique with positive polarity and selected reaction monitoring was used to monitor the transitions *m/z* 380.8 → 100.0 for DR-6180 and *m/z* 373.7 → 100.0 for IS, respectively. The temperature of the ion source capillary was set to 380 °C. The ionization potential was set to 4000 V, the time of scan was 0.3 s and the tube lens was set to 100 V. Peak widths were 0.7 both for Q1 and Q3 quadrupole, the scan width was 0.002 *m/z*. The collision energy was 32 V for both compounds, the pressure of argon in the collision cell was 1.5 mTorr. The gas flows were set to 60, 20, and 25 arb. units for sheath, sweep and auxiliary gas, respectively. The ion source position C was selected.

The injection technique was push-loop using a 20 μL loop, sample viscosity was set to high. The wash solution in the autosampler was methanol, the wash volume was 600 μL. The run time was 8.5 minutes.

The data were evaluated using the Xcalibur program. The concentrations in all samples analyzed in one series were calculated using the same calibration curve parameters. The calibration curves were obtained by weighted linear regression (weighing factor 1/x2): the peak area ratio (analyte/internal standard) was plotted vs. analyte concentration in ng/mL.

*Method optimization*

The response of DR-6180 was further optimized by tuning various parameters of the mass spectrometer (see the Chapter Chromatographic and Mass Spectrometric Conditions). The concentration of formic acid in the aqueous portion of the mobile phase was lowered to 0.1% to increase the sensitivity of detection.

It was observed that during the analytical sequence the peak area of the internal standard was decreasing in consecutive injections, probably due to matrix effects of contaminants from previous runs. Therefore, the gradient program was slightly modified to wash these contaminants from the column with pure methanol.

**References**

[1] O.G. Brakstad, K. Aasbakk, J.A. Maeland, J Clin Microbiol, 30 (1992) 1654-1660.

[2] M.W. Pfaffl, Nucleic Acids Res, 29 (2001) e45.
